# A Phase II Trial of the Epothilone B Analog Ixabepilone (BMS-247550) in Patients with Metastatic Melanoma

**DOI:** 10.1371/journal.pone.0008714

**Published:** 2010-01-20

**Authors:** Patrick A. Ott, Anne Hamilton, Amanda Jones, Naomi Haas, Tsiporah Shore, Sandra Liddell, Paul J. Christos, L. Austin Doyle, Michael Millward, Franco M. Muggia, Anna C. Pavlick

**Affiliations:** 1 Department of Medical Oncology, New York University Cancer Institute, New York, New York, United States of America; 2 Royal Prince Alfred Hospital, Sydney Cancer Centre, Sydney, Australia; 3 Sydney Melanoma Unit and University of Sydney, Sydney, Australia; 4 Division of Hematology/Oncology, Abramson Cancer Center, University of Pennsylvania, Philadelphia, Pennsylvania, United States of America; 5 Division of Hematology/Oncology, Weill Cornell Medical College and New York Presbyterian Hospital, New York, New York, United States of America; 6 Division of Biostatistics and Epidemiology, Weill Cornell Medical College, New York, New York, United States of America; 7 Investigational Drug Branch, National Cancer Institute, Bethesda, Maryland, United States of America; 8 Department of Medical Oncology, Charles Gairdner Hospital and University of Western Australia, Perth, Australia; Dalhousie University, Canada

## Abstract

**Background:**

Ixabepilone (BMS-247550), an epothilone B analog, is a microtubule stabilizing agent which has shown activity in several different tumor types and preclinical models in melanoma. In an open label, one-arm, multi-center phase II trial the efficacy and toxicity of this epothilone was investigated in two different cohorts: chemotherapy-naïve (previously untreated) and previously treated patients with metastatic melanoma.

**Methodology/Principal Findings:**

Eligible patients had histologically-confirmed stage IV melanoma, with an ECOG performance status of 0 to 2. Ixabepilone was administered at a dose of 20 mg/m^2^ on days 1, 8, and 15 during each 28-day cycle. The primary endpoint was response rate (RR); secondary endpoints were time to progression (TTP) and toxicity. Twenty-four patients were enrolled and 23 were evaluable for response. Initial serum lactate dehydrogenase (LDH) levels were elevated in 6/11 (55%) of the previously treated and in 5/13 (38%) of the previously untreated patients. No complete or partial responses were seen in either cohort. One patient in the previously treated group developed neutropenia and fatal septic shock. Seventeen patients (8 in the previously untreated group and 9 in the previously treated group) progressed after 2 cycles, whereas six patients (3 in each group) had stable disease after 2–6 cycles. Median TTP was 1.74 months in the previously untreated group (95% CI = 1.51 months, upper limit not estimated) and 1.54 months in the previously treated group (95% CI = 1.15 months, 2.72 months). Grade 3 and/or 4 toxicities occurred in 5/11 (45%) of previously untreated and in 5/13 (38%) of previously treated patients and included neutropenia, peripheral neuropathy, fatigue, diarrhea, and dyspnea.

**Conclusions/Significance:**

Ixabepilone has no meaningful activity in either chemotherapy-naïve (previously untreated) or previously treated patients with metastatic melanoma. Further investigation with ixabepilone as single agent in the treatment of melanoma is not warranted.

**Trial registration:**

Clinical Trials.gov NCT00036764

## Introduction

There is an urgent need for the identification of active agents in metastatic melanoma. In addition to dacarbazine, temozolomide, and the platinum analogs, the taxanes have shown activity in metastatic melanoma, with overall response rates (RR) in the range of 12%–17% when used as single agents [1], [2], [3], [4], [5], [6], [7]. The epothilones are naturally occurring macrolides produced by the myxobacteria *Sorangium cellulosum*. Like taxanes, their mechanism of action involves the stabilization of microtubules that are necessary for DNA replication and cell division. The exact binding sites on microtubules of taxanes and epothilones overlap but are not identical; however, the microtubule polymerization activity of epothilone B is higher compared to paclitaxel [8]. *In vitro* studies have demonstrated that epothilones have more potent growth inhibition of human prostate, breast, lung, colon, and bladder carcinoma cell lines than the taxanes [9]. An even more marked sensitivity to epothilone B relative to paclitaxel was recently shown in two human melanoma cell lines [10]. Furthermore, the epothilone sagupilone has demonstrated superior efficacy compared to paclitaxel and temozolomide in a mouse CNS metastasis model with MDA-MB-435 melanoma [11]; another epothilone, patupilone resulted in tumor regression in a mouse B16 melanoma model [12].

Ixabepilone (BMS-247550), a semi-synthetic analog of the natural product epothilone B, has been examined in several phase II clinical trials including patients with hormone refractory prostate cancer [13], [14], non-small lung cancer [15], and head and neck cancer [16], amongst others. It was recently approved by the FDA for the treatment of taxane-refractory metastatic breast cancer after a phase III trial showed a significantly longer median time to progression when used in combination with capecitabine compared to capecitabine alone [17]. Adverse events of ixabepilone observed in these studies included hematological toxicities, sensory neuropathy, myalgia, arthralgia, fatigue and diarrhea.

These preclinical and clinical observations provided the rationale to initiate a phase II trial of ixabepilone to assess its efficacy in the treatment of metastatic melanoma.

## Results

### Participant Flow

**Figure 1:**
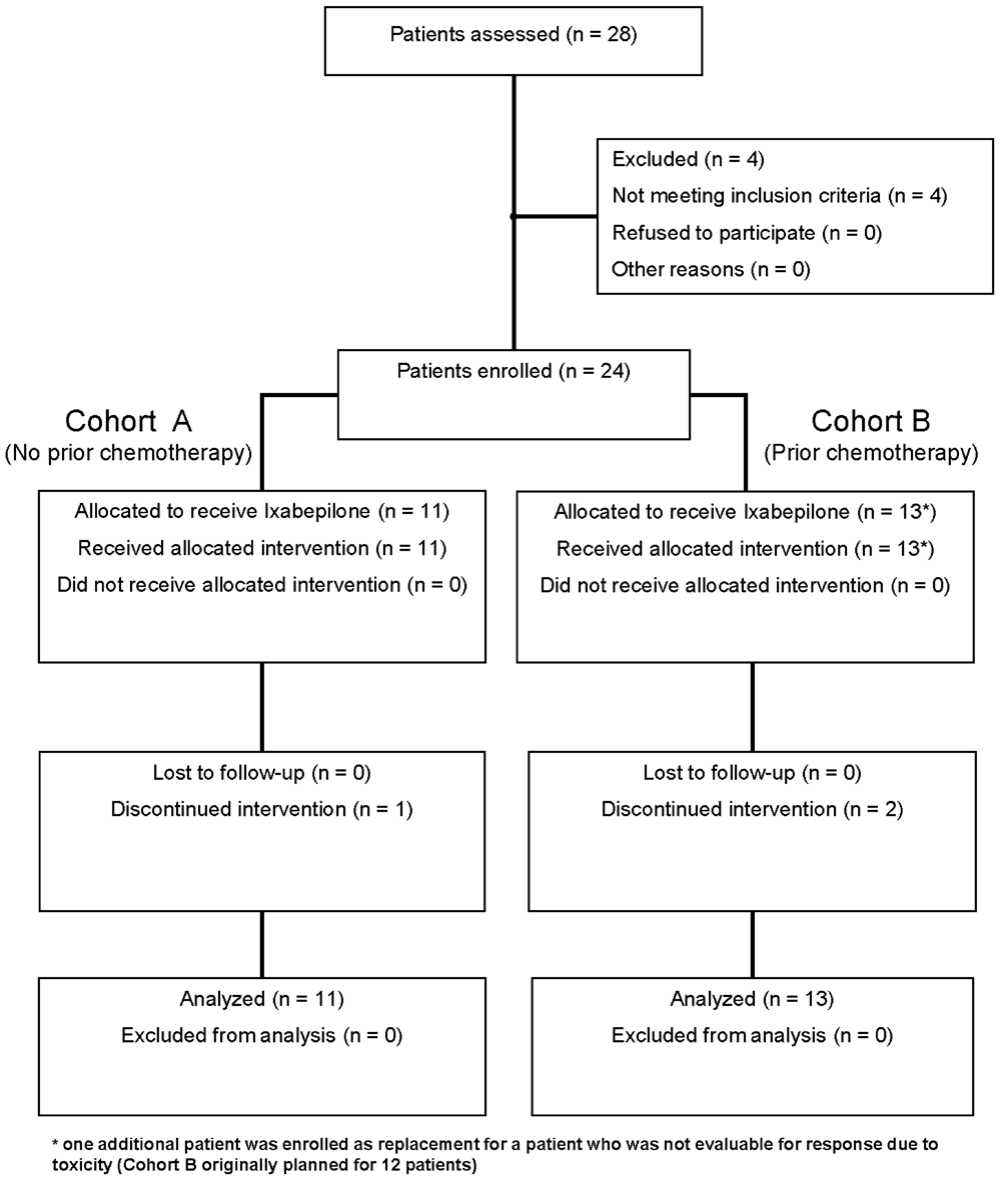
Consort diagram.

The flow of participants through each stage of the study is illustrated in Fig. 1.

One patient had no follow-up disease status evaluation due to death from septic shock after the first cycle of treatment.

### Recruitment

Between March of 2002 and October of 2003, 24 patients were enrolled at 5 centers in the United States and Australia. Patients were followed until disease progression or discontinuation of treatment due to unacceptable side effects, intercurrent illness, or patient withdrawal.

### Baseline Data

Pre-treatment characteristics of the study population are listed in Table 1. All but one patient had an ECOG performance status of 0 or 1. Median age was 55 (range 40–73 years) in the previously untreated patient group and 52 (range 37–62 years) in the previously treated group. Of the 11 previously untreated patients, 6 had primary cutaneous melanoma, one had orbital melanoma, one had ocular melanoma, and 3 had unknown primary melanoma. Of the 13 previously treated patients, 11 had primary cutaneous melanoma and 2 had unknown primary melanoma. Ten of the previously treated patients had received one line of prior chemotherapy and 3 had received 2 lines. All patients with known primary tumor had undergone resection of the tumor. Five of eleven (45%) of previously untreated and 8/13 (62%) of previously treated patients were stage M1c. All patients in the previously treated group had been treated with single agent dacarbazine or temozolomide.

**Table 1:** Patient demographics and disease characteristics.

	1Cohort A			2Cohort B		
Parameter	No. of Patients		%	No. of Patients		%
Enrolled Total	11		100	13		100
Sex						
Male	7		64	8		62
Female	4		36	5		38
Age, Years						
Median		55			52	
Range		40–73			37–62	
Race						
Caucasian	10		91	13		100
Asian	1		9	0		0
ECOG Performance Status						
0	5		45	7		54
1	6		55	5		38
2	0		0	1		8
LDH						
Within Normal Limits	5		45	8		62
Elevated	6		55	5		38
M-Stage						
M1a	2		18	2		15
M1b	4		36	3		23
M1c	5		45	8		62
Prior Immunotherapy	5		45	5		38
Prior Chemotherapy	0		0	13		100
Single Agent				10		77
Multiple Agents				3		23
Prior Radiotherapy	3		27	8		62

1 Previously untreated.

2 previously treated.

### Numbers Analyzed

Eleven patients were enrolled in the previously untreated group. In the previously treated group, one additional patient as replacement for a patient who was not evaluable for response due to toxicity and death from neutropenic sepsis was enrolled, for a total of 13 (instead of the planned 12).

### Outcomes and Estimation

**Figure 2:**
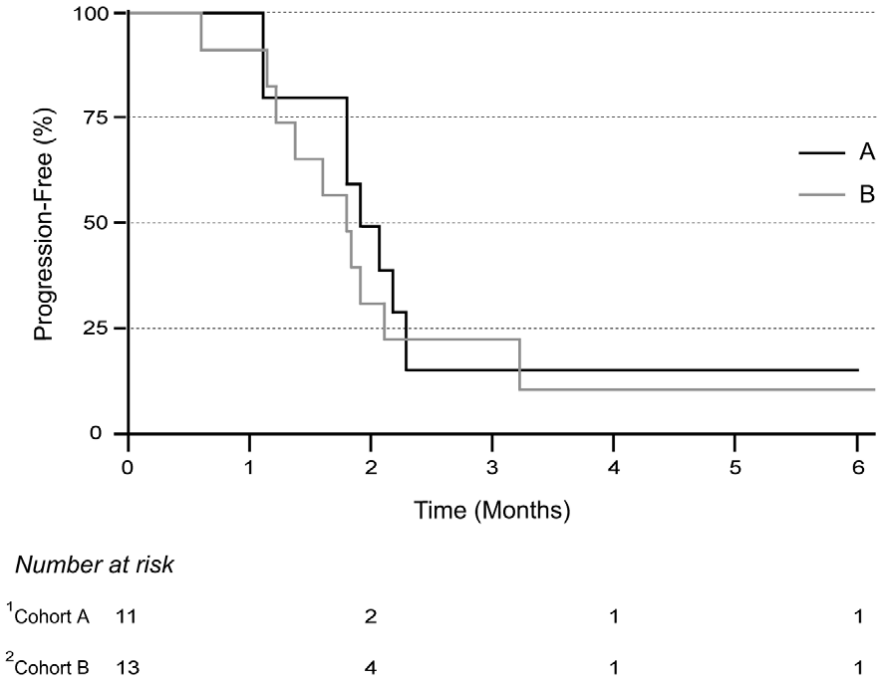
Kaplan-Meier analysis of time to progression. ^1^Cohort A: no previous chemotherapy; ^2^Cohort B: up to two prior chemotherapeutic regimens.

A total of 59 cycles of chemotherapy was administered during the study; the median number of cycles was 2 (range 1–6) in both patient groups. All patients in both subgroups completed at least one cycle of treatment. Treatment was discontinued for disease progression in 73% of patients in the previously untreated cohort and in 69% of patients in the previously treated subgroup. All 11 patients in the previously untreated group and 12 of the 13 patients in the previously treated group were assessable for response, whereas all patients in both groups were assessable for toxicity. No complete response (CR) or partial response (PR) were observed in either patient group (95% confidence intervals: 0–28 for the previously untreated group, 0–26 for the previously treated group, Table 2). Three patients had stable disease (SD) for 2–6 cycles in each cohort, respectively. Median TTP was 1.74 months in the previously untreated group (95% CI = 1.51 months, upper limit not estimated) and 1.54 months in the previously treated group (95% CI = 1.15 months, 2.72 months) (Fig 2). One patient with SD in each group stopped treatment due to grade 3 peripheral neuropathy: one after 3 cycles (previously untreated) and the other one after 2 cycles (previously treated). One patient in the previously treated group who had SD refused further treatment after completing 3 cycles. He had not experienced any significant adverse events during treatment. The remaining 2 patients in the previously untreated group completed six cycles without significant adverse events; one had SD in lung and muscle lesions, while the other had stable lung and skin lesions as well as hilar and abdominal lymphadenopathy, respectively.

**Table 2:** Summary of treatment and responses.

	1Cohort A			2Cohort B		
No of courses administered						
Median	2			2		
Range	1–6			1–6		
	No. of patients	%	95% CI	No. of patients	%	95% CI
Assessable for response (n)	11	100		12	100	
Complete Response	0	0	0–28	0	0	0–26
Partial Response	0	0	0–28	0	0	0–26
Stable disease	3	27		3	25	
Progression of disease	8	73		9	75	

1 Previously untreated.

2 previously treated.

CI: Confidence interval.

According to the stopping rules of the 3-stage design (Table 3), accrual was terminated after stage one, since no responses were seen in either of the 2 cohorts.

**Table 3:** Three-stage patient accrual.

Accrual Stage	Number of Patients to be accrued in Stage	Cumulative Number of Patients accrued at End of Stage	Reject Drug if Response Rate≤r^1^/n
1Cohort A			
1	11	11	0/11
2	18	29	3/29
3	21	50	7/50
2Cohort B			
1	12	12	0/12
2	13	25	1/25
3	13	38	3/38

1 Previously untreated.

2 previously treated.

### Adverse Events

All patients were evaluable for safety. Overall, grade 3 and/or 4 toxicities occurred in 5/11 (45%) of previously untreated and in 5/13 (38%) of previously treated patients. The hematological and non-hematological adverse events experienced by the patients in this trial are summarized in Table 4. Grade 3 and 4 neutropenia occurred in 1/11 (9%) of previously untreated and in 3/13 (23%) of previously treated patients. The predominant non-hematological toxicities were neuropathy, diarrhea, dyspnea, and fatigue in both cohorts. The incidence of neuropathy was 55% (all grades) and 9% (grade 3) in the previously untreated subgroup and 38% (all grades) and 8% (grade 3) in the previously treated subgroup. Treatment had to be terminated for grade 3 neuropathy during cycle 2 for one patient in the previously untreated group and during cycle 3 for one patient in the previously treated cohort. Six of the 11 (55%) patients in the previously untreated group and 4/13 (31%) patients in the previously treated group developed diarrhea, which was generally easily manageable and did not result in treatment termination for any of the patients. Fatigue occurred in 6/11 (55%) of the patients in the previously untreated subgroup and in 9/13 (69%) of the patients in the previously treated subgroup, but was grade 3 in only one patient in the previously untreated group and did not lead to treatment cessation. There was one death in which treatment was implicated. The patient, who was in the previously treated group of the study, developed febrile neutropenia, thrombocytopenia, and a suspected stroke after the first cycle of chemotherapy and died during cycle 1 as a result of septic shock.

**Table 4:** Toxicity profile.

	1Cohort A (*n* = 11)					2Cohort B (n = 13)				
	Toxicity grade									
Adverse Event	1	2	3	4	3/4	1	2	3	4	3/4
	Number of patients (%)									
*Hematological*										
Leukopenia	0	1 (9)	0	0	0	0	0	1 (8)	1 (8)	2 (15)
Neutropenia	0	0	1 (9)	0	1 (9)	0	1 (8)	1 (8)	2 (15)	3 (23)
Anemia	1 (9)	1 (9)	1 (9)	0	1 (9)	0	0	0	0	0
Thrombocytopenia	0	0	0	0	0	0	0	0	1 (8)	1 (8)
*Non-hematological*										
Anxiety	0	0	1 (9)	0	1 (9)	0	0	0	0	0
Neuropathy	3 (27)	2 (18)	1 (9)	0	1 (9)	4 (31)	0	1 (8)	0	0
Hypotension	0	0	0	1 (9)	1 (9)	0	0	0	0	0
Arrythmia	0	0	0	1 (9)	1 (9)	0	0	0	0	0
Fatigue	4 (36)	1 (9)	1 (9)	0	1 (9)	4 (31)	5 (38)	0	0	0
Dyspnea	0	0	1 (9)	0	1 (9)	0	2 (15)	1 (8)	0	1 (8)
Diarrhea	1 (9)	1 (9)	4 (36)	0	4 (36)	4 (31)	0	0	0	0
Constipation	0	0	0	0	0	0	0	1 (8)	0	1 (8)
Abdominal distension	0	0	0	0	0	0	0	1 (8)	0	1 (8)
Gastritis	0	1 (9)	0	0	0	0	0	0	0	0
Nausea	4 (36)	2 (18)	0	0	0	0	2 (15)	0	0	0
Anorexia	0	0	0	0	0	0	1 (8)	0	0	0
Pneumonitis	0	0	0	0	0	0	0	1 (8)	0	1 (8)
Infection	0	0	0	0	0	0	1 (8)	0	0	0
Alopecia	2 (18)	0	0	0	0	1 (8)	2 (15)	0	0	0
Rash/desquamation	2 (18)	0	0	0	0	1 (8)	1 (8)	0	0	0

1 Previously untreated.

2 previously treated.

## Discussion

A major interest in clinical trials has been the efficacy of epothilones in taxane-resistant cancers. The known activity of epothilones in other solid tumors provided the rationale to characterize the activity of an epothilone in metastatic melanoma patients.

This was the first trial conducted assessing the activity of ixabepilone in melanoma patients. Ixabepilone as a single agent did not show activity in the group of patients with metastatic melanoma enrolled in our study. The absence of an objective response was disappointing since taxanes used as single agents do have activity in metastatic melanoma with RR comparable to dacarbazine in phase II trials. Apart from breast cancer, clinical activity has been demonstrated for ixabepilone in a variety of tumor types with RR generally in the 10–20% range [13], [14], [15], [16], [18], [19], [20]. Gene expression levels of the microtubule associated protein *tau* have recently been described as inversely correlated with response to epothilones in breast cancer patients [21]. We speculate that *tau* expression levels might be higher in advanced melanoma patients as compared to other solid tumors.

The major toxicities of ixabepilone in this trial were neutropenia, peripheral neuropathy, diarrhea, dyspnea, and fatigue. Two patients (8%) discontinued protocol therapy because of grade 3 neuropathy. Neuropathy has been a prominent side effect in previous phase II and phase III trials with a frequency of grade 3 neuropathy in the 5–20% range [14], [15], [16], [22], [23], [24]. Furthermore, one patient died from neutropenic sepsis. A relatively high degree of bone marrow suppression has been reported previously with the use of ixabepilone as a single agent [19], [25].

The high incidence of grade 3/4 neuropathy and myelosuppression is troubling. In retrospect, the optimal dosing protocol for ixabepilone had not yet been fully established at the time of this trial design. The agent is most commonly given at 40 mg/m^2^ on a 3-weekly schedule, but protocols have also used lower doses given for several days on a 3-week cycle. A dose of 20 mg/m^2^ on a weekly schedule for 3 weeks of a 4 week cycle was established in a phase I dose escalation study; it was this dose schedule that was recommended for phase II trials in cancers with no established standard chemotherapy [26] and was chosen for this study. The median number of treatment cycles administered to patients in this study was lower than in other single agent ixabepilone trials (2 vs. 3–5), suggesting that neuropathy and myelosuppression could be underestimated compared to other studies. This would suggest a higher toxicity of the weekly schedule, which has been associated previously with an increased rate of side effects compared to every 3 week regimens [16].

In conclusion, ixabepilone as a single agent has no detected activity in patients with metastatic melanoma and has a relatively severe toxicity profile compared to agents currently in use for this disease such as temozolomide, dacarbazine, paclitaxel, and carboplatin. Based on these data, further development of ixabepilone as monotherapy is not warranted in patients with metastatic melanoma. Given the documented activity of microtubule stabilizing agents in melanoma and the preclinical data documenting efficacy of epothilones in melanoma cell lines, it is reasonable to project that newer-generation epothilones, as they are being developed [27], might still have a role in the future treatment of melanoma.

## Methods

The protocol for this trial and supporting CONSORT checklist are available as supporting information; see Checklist S1 and Protocol S1.

### Ethics

This study was conducted according to the principles expressed in the Declaration of Helsinki. The protocol (NCI study number NCI-4470, Clinical Trials.gov identifier NCT00036764) received prior approval by the institutional review board at New York University Langone Medical Center. The protocol was reviewed by the local institutional review board at each participating institution, and all patients provided written informed consent.

### Participants

Eligible patients had histologically or cytologically confirmed metastatic melanoma, with an Eastern Cooperative Oncology Group (ECOG) performance status of 0 to 2. Life expectancy of greater than 3 months was required, and patients were at least 18 years of age. All patients had measurable disease according to the international criteria proposed by the Response Evaluation Criteria in Solid Tumors (RECIST) committee [28]. Two different subgroups were studied in a single arm phase II study: 1) patients who were chemotherapy naïve (previously untreated) and 2) patients who had previously received a maximum of two prior lines of chemotherapy with mandatory dacarbazine or temolozomide (previously treated). Patients with known brain metastases were included in the study if they were steroid independent with radiographically stable lesions for at least six weeks after whole brain radiation and no mass effect present radiographically at the time of study entry. Other eligibility criteria were normal laboratory values (absolute neutrophil count of ≥1.5×10^9^/L, platelets ≥100×10^9^/L, total bilirubin within normal institutional limits, AST and ALT ≤2.5 times the upper limits of normal, and creatinine ≤1.5 times the upper limit of normal). All women of childbearing age had to agree to use contraception prior to study entry and for the duration of study; pregnant women were excluded. Patients who were receiving any other investigational agents were excluded from the study. Exclusion criteria also consisted of a history of severe allergic reactions (grade III or IV or grade II not responsive to corticosteroids) attributed to medications containing Cremophor. Patients with pre-existing grade II–IV peripheral neuropathy were excluded. Other exclusion criteria comprised patients with uncontrolled concomitant illness including but not limited to ongoing or active infection, HIV+ on anti-retroviral therapy, symptomatic congestive heart failure, unstable angina pectoris, cardiac arrhythmia, or psychiatric illness that would limit compliance with study requirements.

The study was conducted by the New York Cancer Consortium (www.newyorkcancerconsortium.org). The participating institutions were New York University Langone Medical Center, New York, NY, New York Presbyterian Hospital, New York, NY, Fox Chase Cancer Center, Philadelphia, PA, Austin, Repatriation Medical Centre, Melbourne, Australia, and Sydney Cancer Center, Sydney, Australia. The study was reviewed, approved, and sponsored by the Cancer Therapy Evaluation Program of the National Cancer Institute.

### Interventions

Ixabepilone was evaluated as a single agent in patients with stage IV malignant melanoma. All patients were treated with ixabepilone at 20 mg/m^2^ administered as a 1 hour infusion on days 1, 8, and 15 of each 28-day cycle. This dosing regimen (rather than the more commonly used 40 mg/m^2^ every 3 weeks) was chosen based on phase I data in patients with advanced malignancy with no standard treatment options [26]. Patients received premedication with diphenhydramine 50 mg i.v., ranitidine 50 mg i.v., ondansetron 8 mg i.v./po., and dexamethasone 20 mg i.v. to minimize nausea, vomiting, and hypersensitivity reactions. Treatment was stopped at any time point due to disease progression or intolerable side effects. Adverse events were reported using the revised NCI Common Toxicity Criteria (CTC) version 3.0. Hematologic growth factors were not used prophylactically in this study, but were used at the discretion of the investigator in the event of severe hematologic toxicity.

### Objectives

The objectives of this phase II trial were to assess the efficacy of ixabepilone in metastatic melanoma patients and to expand upon the known toxicity profile of ixabepilone at the recommended phase II dose. The hypothesis, based on preclinical data in melanoma cell lines and a mouse model, was that ixabepilone has efficacy in metastatic melanoma patients.

### Outcomes

The primary endpoint was objective RR, while time to tumor progression (TTP) and toxicity were assessed as secondary endpoints. Response and progression were evaluated in this study using RECIST criteria [28]. Complete history and physical examination, assessment of ECOG performance status, routine laboratory studies and appropriate imaging studies to evaluate the extent of metastatic disease were performed at enrollment. Patients were assessable for response if they received one or more cycles of treatment; treatment response was evaluated every two cycles using appropriate radiographic imaging studies, complete history and physical examinations, and routine laboratory studies. Time to progression was defined as the time from the first day of treatment with ixabepilone until the first documentation of disease progression. For patients who did not progress, the date of last follow-up was used to censor the patients at that point. Patients were treated until disease progression or development of unacceptable toxicities.

### Sample Size and Statistical Design

The three-stage optimal design for phase II clinical trials proposed by Ensign et al. [29] was used in each subgroup (group A: previously untreated, group B: previously treated with chemotherapy). We projected that ixabepilone would have a RR of 10% in the previously untreated and 5% in the previously treated group, below which the response would be unacceptable, and a RR of 25% in the previously untreated and 20% in the previously treated group, above which the regimen would be considered worthy of further exploration. The null hypothesis that the overall response proportion would be less than or equal to 10% (for the previously untreated group) and 5% (for the previously treated group) was tested against the alternative hypothesis that the response proportion would be greater than or equal to 25% (for the previously untreated group) and 20% (for the previously treated group).

The statistical design and planned 3-stage patient accrual, including the numbers of patients to be accrued at each stage in both subgroups is described in Table 3. A total sample size of 23–88 evaluable patients was planned. Using this design, both the alpha and beta error probabilities were 0.10 for both cohorts. The alpha level being used was one-sided. A beta error probability of 0.10 (lower than the commonly used 0.20) was chosen because of the paucity of new drugs that may be active in melanoma. No formal comparison between the 2 subgroups was planned or performed.

Descriptive statistics and percentages are presented for demographic and clinical characteristics. TTP was analyzed using the Kaplan-Meier method. Ninety-five percent confidence intervals (95% CI) for median TTP and the observed RR were calculated to assess the precision of the obtained estimates. All analyses were performed in SAS version 9.1 (SAS Institute, Inc., Cary, NC) and Stata version 10.0 (Stata Corporation, College Station, TX).

## Supporting Information

Protocol S1 Trial Protocol.
(0.55 MB DOC)

Checklist S1 CONSORT Checklist.
(0.19 MB DOC)

## References

[1] Aamdal S, Wolff I, Kaplan S, Paridaens R, Kerger J (1994). Docetaxel (Taxotere) in advanced malignant melanoma: a phase II study of the EORTC Early Clinical Trials Group.. Eur J Cancer.

[2] Bedikian AY, Plager C, Papadopoulos N, Eton O, Ellerhorst J (2004). Phase II evaluation of paclitaxel by short intravenous infusion in metastatic melanoma.. Melanoma Res.

[3] Bedikian AY, Weiss GR, Legha SS, Burris HA, Eckardt JR (1995). Phase II trial of docetaxel in patients with advanced cutaneous malignant melanoma previously untreated with chemotherapy.. J Clin Oncol.

[4] Einzig AI, Hochster H, Wiernik PH, Trump DL, Dutcher JP (1991). A phase II study of taxol in patients with malignant melanoma.. Invest New Drugs.

[5] Einzig AI, Schuchter LM, Recio A, Coatsworth S, Rodriquez R (1996). Phase II trial of docetaxel (Taxotere) in patients with metastatic melanoma previously untreated with cytotoxic chemotherapy.. Med Oncol.

[6] Gogas H, Bafaloukos D, Bedikian AY (2004). The role of taxanes in the treatment of metastatic melanoma.. Melanoma Res.

[7] Legha SS, Ring S, Papadopoulos N, Raber M, Benjamin RS (1990). A phase II trial of taxol in metastatic melanoma.. Cancer.

[8] Kowalski RJ, Giannakakou P, Hamel E (1997). Activities of the microtubule-stabilizing agents epothilones A and B with purified tubulin and in cells resistant to paclitaxel (Taxol(R)).. J Biol Chem.

[9] Altmann KH, Wartmann M, O'Reilly T (2000). Epothilones and related structures–a new class of microtubule inhibitors with potent in vivo antitumor activity.. Biochim Biophys Acta.

[10] Azzabi A, Hughes AN, Calvert PM, Plummer ER, Todd R (2005). Phase I study of temozolomide plus paclitaxel in patients with advanced malignant melanoma and associated in vitro investigations.. Br J Cancer.

[11] Hoffmann J, Fichtner I, Lemm M, Lienau P, Hess-Stumpp H (2008). Sagopilone crosses the blood-brain barrier in vivo to inhibit brain tumor growth and metastases.. Neuro Oncol.

[12] Ferretti S, Allegrini PR, O'Reilly T, Schnell C, Stumm M (2005). Patupilone induced vascular disruption in orthotopic rodent tumor models detected by magnetic resonance imaging and interstitial fluid pressure.. Clin Cancer Res.

[13] Galsky MD, Small EJ, Oh WK, Chen I, Smith DC (2005). Multi-institutional randomized phase II trial of the epothilone B analog ixabepilone (BMS-247550) with or without estramustine phosphate in patients with progressive castrate metastatic prostate cancer.. J Clin Oncol.

[14] Hussain M, Tangen CM, Lara PN, Vaishampayan UN, Petrylak DP (2005). Ixabepilone (epothilone B analogue BMS-247550) is active in chemotherapy-naive patients with hormone-refractory prostate cancer: a Southwest Oncology Group trial S0111.. J Clin Oncol.

[15] Vansteenkiste J, Lara PN, Le Chevalier T, Breton JL, Bonomi P (2007). Phase II clinical trial of the epothilone B analog, ixabepilone, in patients with non small-cell lung cancer whose tumors have failed first-line platinum-based chemotherapy.. J Clin Oncol.

[16] Burtness BA, Manola J, Axelrod R, Argiris A, Forastiere AA (2008). A randomized phase II study of ixabepilone (BMS-247550) given daily×5 days every 3 weeks or weekly in patients with metastatic or recurrent squamous cell cancer of the head and neck: an Eastern Cooperative Oncology Group study.. Ann Oncol.

[17] Thomas ES, Gomez HL, Li RK, Chung HC, Fein LE (2007). Ixabepilone plus capecitabine for metastatic breast cancer progressing after anthracycline and taxane treatment.. J Clin Oncol.

[18] Ajani JA, Safran H, Bokemeyer C, Shah MA, Lenz HJ (2006). A multi-center phase II study of BMS-247550 (Ixabepilone) by two schedules in patients with metastatic gastric adenocarcinoma previously treated with a taxane.. Invest New Drugs.

[19] Dreicer R, Li S, Manola J, Haas NB, Roth BJ (2007). Phase 2 trial of epothilone B analog BMS-247550 (ixabepilone) in advanced carcinoma of the urothelium (E3800): a trial of the Eastern Cooperative Oncology Group.. Cancer.

[20] Posadas EM, Undevia S, Manchen E, Wade JL, Colevas AD (2007). A phase II study of ixabepilone (BMS-247550) in metastatic renal-cell carcinoma.. Cancer Biol Ther.

[21] Baselga J, Zambetti M, Llombart-Cussac A, Manikhas G, Kubista E (2009). Phase II genomics study of ixabepilone as neoadjuvant treatment for breast cancer.. J Clin Oncol.

[22] Goel S, Goldberg GL, Kuo DY, Muggia F, Arezzo J (2008). Novel neurosensory testing in cancer patients treated with the epothilone B analog, ixabepilone.. Ann Oncol.

[23] Perez EA, Lerzo G, Pivot X, Thomas E, Vahdat L (2007). Efficacy and safety of ixabepilone (BMS-247550) in a phase II study of patients with advanced breast cancer resistant to an anthracycline, a taxane, and capecitabine.. J Clin Oncol.

[24] Roche H, Yelle L, Cognetti F, Mauriac L, Bunnell C (2007). Phase II clinical trial of ixabepilone (BMS-247550), an epothilone B analog, as first-line therapy in patients with metastatic breast cancer previously treated with anthracycline chemotherapy.. J Clin Oncol.

[25] Eng C, Kindler HL, Nattam S, Ansari RH, Kasza K (2004). A phase II trial of the epothilone B analog, BMS-247550, in patients with previously treated advanced colorectal cancer.. Ann Oncol.

[26] Awada A, Piccart MJ, Jones SF, Peck RA, Gil T (2009). Phase I dose escalation study of weekly ixabepilone, an epothilone analog, in patients with advanced solid tumors who have failed standard therapy.. Cancer Chemother Pharmacol.

[27] Chou TC, Zhang X, Zhong ZY, Li Y, Feng L (2008). Therapeutic effect against human xenograft tumors in nude mice by the third generation microtubule stabilizing epothilones.. Proc Natl Acad Sci U S A.

[28] Therasse P, Arbuck SG, Eisenhauer EA, Wanders J, Kaplan RS (2000). New guidelines to evaluate the response to treatment in solid tumors. European Organization for Research and Treatment of Cancer, National Cancer Institute of the United States, National Cancer Institute of Canada.. J Natl Cancer Inst.

[29] Ensign LG, Gehan EA, Kamen DS, Thall PF (1994). An optimal three-stage design for phase II clinical trials.. Stat Med.

