# Supplementary material for: A Phase II Trial of the Epothilone B Analog Ixabepilone (BMS-247550) in Patients with Metastatic Melanoma
Source: PLoS One. 2010 Jan 20;5(1):e8714. doi: 10.1371/journal.pone.0008714 (PMC2808339; doi:10.1371/journal.pone.0008714)
Supplement: Protocol S1 — Trial Protocol. (0.55 MB DOC) [file pone.0008714.s001.doc]

NYU 00-57

**NCI Protocol #: 4470**

A Phase II Study of Epothilone B Analog
BMS 247550 (NSC # 710428) in stage IV Malignant Melanoma

**Coordinating Center:** **New York University School of Medicine**

**Kaplan Comprehensive Cancer Center**

**Protocol Chair:** **Anna Pavlick, DO**

#### NYU School of Medicine

Bellevue C&D Building, Room 556

462 First Ave

New York, NY 10016

USA

Tel: 1 (212) 263 6485

e-mail: anna.pavlick@med.nyu.edu

**Co-Investigators /** **Franco Muggia, MD**

**Participating Institutions: Anne Hamilton, MBBS**

**Iman Osman,MD**

NYU School of Medicine

**Sridhar Mani, MD**

**Dr Tsiporah Shore, MD**

New York Presbyterian

Weill Medical College of Cornell University

520 East 70^th^ Street

Starr-341

New York, NY 10021

Tel: (212) 746-2646

Email: [tbs2001@med.cornell.edu](mailto:tbs2001@med.cornell.edu)

### Naomi Haas, MD

Fox Chase Cancer Center

Dept. of Medical Oncology

Fox Chase Cancer Center

7701 Burholme Ave.

Philadelphia, PA 19095 USA

Tel: 1 (215) 728 2974

e-mail: NHaas@exchserver.fccc.edu

***February 21, 2003***

**Jonathan Cebon, MBBS, PhD**

Ludwig Institute for Cancer Research

Austin and Repatriation Medical Centre

Studley Rd

Heidelberg, VIC 3084

Australia

Tel: 61 (3) 9457 6933

Email: [jonathan.cebon@ludwig.edu.au](mailto:jonathan.cebon@ludwig.edu.au)

**Peter Gibbs, MBBS**

The Royal Melbourne Hospital

C/O Post Office

Parkville 3050

Australia

Tel: 61 (3) 9342 4269

E-mail: peter.gibbs@mh.org.au

##### Michael Millward, MBBS

Sydney Cancer Centre

Royal Prince Alfred Hospital

Gloucester House

Missenden Rd

Camperdown, NSW 2050

Australia

Tel: 61 (2) 9515 7680

Email: [michaelm@canc.rpa.cs.nsw.gov.au](mailto:michaelm@canc.rpa.cs.nsw.gov.au)

**Correlative Studies:** **Susan Horwitz, PhD**

**Hayley McDaid, PhD**

Albert Einstein College of Medicine
Molecular Pharmacology

Golding, Rm 201

1300 Morris Pk Ave

Bronx, NY 10461

Tel: 1 (718) 430 2192

Email:shorwitz@aecom.yu.edu

[mcdaid@aecom.yu.edu](mailto:mcdaid@aecom.yu.edu)

**Statistician:** **Anne Jacquotte, MD MS**

NYU School of Medicine

OPH

341 East 25th Street,

New York, NY 10010

Tel: 1 (212) 263 6512

e-mail: jacqua02@ med.nyu.edu

**Study Coordinator:** **Amanda Bailes**

#### NYU School of Medicine

Bellevue C&D Building, Room 556

462 First Ave

New York, NY 10016

USA

Tel: 1 (212) 263 6485

Fax: 1 (212) 263 8210

e-mail: bailea02@gcrc.med.nyu.edu

**NCI-Supplied Agent**: Epothilone B Analog (BMS 247550; NSC 710428)

**SCHEMA**

Two subgroups of patients will be treated:

**Subgroup A: Chemotherapy naïve**

These patients may not have received any prior chemotherapy

**Subgroup B: DTIC pretreated**

These patients may have received a maximum of two prior lines of chemotherapy, and must have received DTIC or temozolomide.

All patients will be treated with Epothilone B analogue BMS 247550, 20 mg/m2 IV over 1 hour on days 1,8,15 of a 28 day cycle.

**TABLE OF CONTENTS**

Page

**SCHEMA**  4

**1. OBJECTIVES**  7

**2. BACKGROUND**  7

2.1 **Malignant Melanoma Stage IV**  8

2.2 **Epothilone B Analog (BMS 247550)**  8

2.3 **Rationale**  18

**3. PATIENT SELECTION**  18

3.1 **Eligibility Criteria** 18

3.2 **Exclusion Criteria**  19

3.3 **Inclusion of Women and Minorities**  20

**4. TREATMENT PLAN**  20

4.1 **Agent Administration**  20

4.2 **Supportive Care Guidelines**  21

4.3 **Duration of Therapy**  23

**5. EXPECTED ADVERSE EVENTS/DOSE MODIFICATIONS**  23

5.1 **Expected Adverse Events Associated with BMS 247550**  23

5.2 **Dosing Delays/Dose Modifications**  24

**6. AGENT FORMULATION AND PROCUREMENT**  24

**7. CORRELATIVE/SPECIAL STUDIES**  27

**8. STUDY CALENDAR**  30

**9. MEASUREMENT OF EFFECT**  31

9.1 **Definitions**  31

9.2 **Guidelines for Evaluation of Measurable Disease**  32

9.3 **Response Criteria**  33

9.4 **Confirmatory Measurement/Duration of Response**  34

9.5 **Progression-Free Survival**  35

9.6 **Response Review**  35

**10. REGULATORY AND REPORTING REQUIREMENTS** 35

10.1 **Patient Registration** 35

10.2 **Expedited Adverse Event Reporting** 36

10.3 **Data Reporting**  37

10.4 **CTEP Multicenter Guidelines**  37

10.4 **Clinical Trials Agreement (CTA)** ………………………………………………….38

**11. STATISTICAL CONSIDERATIONS**  39

**12. REFERENCES** 42

**MODEL INFORMED CONSENT FORM**

**APPENDICES**

##### APPENDIX A

Expected Adverse Events Associated With BMS 247550 A-1

##### APPENDIX B

Performance Status Criteria B-1

##### APPENDIX C

Specimen Collection Instructions..……………………………………………………...C-1

##### APPENDIX D

Eligibility Criteria…..…………………………………………………………………...D-1

##### APPENDIX E

Sample Forms… ………………………………………………………………………..E -1

1. OBJECTIVES

1.1 To assess the efficacy of BMS 247550 in stage IV malignant melanoma

1.2 To expand upon the known toxicity profile of BMS 247550 at the recommended phase II dose

1.3 To explore whether there is an association between pharmacokinetics at the recommended phase II doses with the extent of microtubule bundle and mitotic aster formation in peripheral blood mononuclear cells and tumor cells, where available. Total RNA, genomic DNA and total protein will also be extracted and stored from these cells for subsequent molecular analyses.

**2. BACKGROUND**

**2.1 Malignant Melanoma Stage IV**

Melanoma is an increasingly important health problem. The incidence of melanoma has increased at a rate of 4% per year over the last two decades with rates now approaching 30 per 100,000 in some populations (1). Surgery can be curative in Stage I, II, or III disease, but a large number of patients with deep primary lesions or nodal involvement will develop extensive recurrence or distant metastases (stage IV disease). No curative treatment exists for stage IV melanoma. Dacarbazine (DTIC) or DTIC-containing regimens are the most commonly used treatments for advanced disease.

## 2.1.1 Standard Therapy

In the first-line chemotherapy treatment of patients with stage IV disease, agents with reproducible activity against melanoma include DTIC, cisplatin, nitrosoureas and vinca alkaloids. DTIC is the most active single agent with response rates ranging from about 10% to 20%, and median response durations of 4 to 6 months (2). Although several recent studies using a combination of DTIC and other agents have shown increased response rates (see below), these combinations have not proven to be superior to single agent DTIC for the general population. Similarly, a Phase III study comparing temozolomide to DTIC showed no substantial improvement in survival or in other primary clinical endpoints (3).

A variety of combination chemotherapy regimens have produced response rates of 30% to 50% in single-institution Phase 2 trials. Two of the more active regimens were the 3-drug combination of cisplatin/vinblastine/DTIC (CVD) (4) and the 4-drug combination of cisplatin/DTIC/BCNU/tamoxifen (CDBT) (5). However, a randomized multi-institutional trial comparing CVD to DTIC alone, the CVD arm was not significantly superior in response rate, response duration, or survival (6). In a recent update of this trial, that encompassed approximately 150 patients, the CVD arm produced a 19% response rate compared to 14% for DTIC alone with no difference in either response duration or survival. A randomized, Phase 3 trial (EST 91-140) also demonstrated no significant survival benefit associated with CDBT relative to DTIC alone.

Several recent studies have indicated potential value for the addition of either interferon-α or tamoxifen to DTIC (7-9). The actual benefit of the addition of interferon and/or tamoxifen to DTIC in patients with advanced melanoma was tested by the Eastern Cooperative Oncology Group (ECOG) in a large-scale, four-arm, Phase 3 trial (EST 3690). The overall response rate was 18% (range 12% to 21% for the 4 arms), median time to treatment failure was 2.6 months, and median survival was 9.1 months. There was no increase in objective response, increased duration of time to progression, or survival advantage attributable to the addition of interferon, tamoxifen, or both to DTIC. Based on this trial and the cumulative data from prior studies, there is no compelling evidence to support the addition of either interferon or tamoxifen to DTIC in this disease.

## 2.1.2 Immunotherapy

A variety of clinical and laboratory observations have suggested that host immunologic mechanisms may occasionally influence the course of melanoma which have fostered interest in the use of biologic response modifiers. During the past decade, two biologic agents, interferon-α (IFN-α) and IL-2, have shown reproducible single agent antitumor activity against advanced melanoma. Both IL-2 and IFN-α have produced response rates in the 15 to 20% range (10,11). High dose IL-2 therapy, administered by intravenous bolus either alone or in combination with LAK cells, has produced durable complete responses in approximately 5% of patients (11-14). However, the known adverse effects of IL-2 have precluded wide application to patients with common medical conditions due to the increased risk of treatment-related morbidity.

## 2.1.3 Biochemotherapy Combinations

A number of investigators have studied combinations of cytotoxic chemotherapy with IL-2 based immunotherapy. In general, the best results have been observed in studies that combined DTIC- and/or cisplatin-based chemotherapy with either high-dose IL-2 alone, or lower doses of IL-2 combined with IFN-α.

Ongoing Phase 2 and 3 trials include CVD ± IL-2/IFN administered in a sequential fashion (M.D. Anderson Cancer Center), cisplatin/DTIC ± IL-2/IFN (NCI Surgery Branch), and E3695, the intergroup (ECOG/SWOG/CALGB) study of CVD ± IL-2/IFN. While the results of these studies will be important for patients with good performance status, the toxicity of these regimens will limit their applicability to the general population.

New agents that are more active against melanoma than platinum and DTIC are clearly desperately required.

**2.2**  **BMS 247550**

Small molecules that bind to tubulin and/or microtubules constitute a large family of compounds with diverse functions such as herbicides, insecticides and antineoplastic compounds The antineoplastics typically bind to tubulin and act as antimitotic agents that block normal mitotic spindle function, the cellular structure that forms mitotic spindles and is required for chromosome segregation (15). Such drugs can either promote tubulin depolymerization in cells (e.g. vinca alkaloids) or promote polymerization of stable microtubules (e.g. taxanes) (16-18). Significant clinical advances have been made with these agents that are now integral components of curative and palliative regimens for several solid tumors including, but not limited to breast, ovarian, lung and other malignancies (16-20).

Microtubules comprise tubulin heterodimers that are composed of related proteins, α- and β-tubulin subunits (each is around 450 amino acids with MW ~50,000). When tubulin heterodimers assemble into microtubules, they form “linear protofilaments” with the β-tubulin of one subunit in contact with the α-tubulin of the next. Microtubules consist of 13 protofilaments aligned in parallel with the same polarity (i.e. one end assembles rapidly while the other has a slower assembly or greater net disassembly of tubulin). γ-tubulin appears to localize to centrosomes. The gene sequence of α- and β-tubulin are highly conserved across species. Additionally, α- and β-tubulin have multiple isotypes which are distinguished by slight differences in amino acid composition at the C-terminus. In addition to isotype distribution, tubulins undergo post-translational modifications, including acetylation, glutamylation and detyrosylation which may also account for functional differences of microtubules in various tissues (21-27). At least six human α- and β-tubulin isotypes have been identified. The six β-tubulin isotypes are distinguished on the basis of differences in C-terminal amino acid composition as well as differences in post-translational modifications including phosphorylation and glutamylation (23-26). Microtubules are dynamic and their polymerization is affected by several factors including GTP (which binds to one exchangeable site on β-tubulin and one non-exchangeable site on α-tubulin); the ionic environment (e.g. Ca^2+^ concentration), and existence of microtubule-associated proteins or MAPs.

Taxol binds to polymerized tubulin resulting in the hyperstabilization of microtubules, even in the absence of factors that are normally essential for this function, such as GTP or microtubule-associated proteins (28). Such microtubules are resistant to depolymerization by calcium or low temperatures. This results in the suppression of microtubule dynamics and the sustained arrest of cells in mitosis, which inhibits proliferation and is associated with programmed cell death (29). Morphological features of cells exposed to paclitaxel include the appearance of stable bundles or parallel arrays of microtubules, and mitotic asters. Using three different photoaffinity analogs of paclitaxel three domains in β-tubulin have been identified that are in contact with the drug (30,31). These studies are in agreement with the electron crystallography model, data published by Nogales et al, in which the α- and β-tubulin dimer is fitted to a 3.7Å density map (27). Recently, other proteins in addition to tubulin have been shown to interact with paclitaxel, including Bcl-2 (31) and CD-18, the approximately 96kDa common component of the β2-integrin family (32).

In 1983, paclitaxel was approved for phase I clinical trials and shown to be active particularly in combination with other cytotoxic agents in the treatment of patients with ovarian, lung and early stage breast carcinomas (19,32). Despite its clinical success, paclitaxel's hydrophobicity and therefore aqueous insolubility has complicated its formulation, and renders the drug a substrate for p-glycoprotein, an energy-dependent drug efflux pump that maintains a low intracellular drug concentration (32,33). Ultimately, the majority of patients eventually develop paclitaxel-resistant disease and many cancer types such as colorectal cancers are intrinsically resistant to this and other related taxanes. There are many possible mechanisms for paclitaxel-induced resistance including mutations in β-tubulin which may abrogate the ability of paclitaxel to bind to its cellular target, alterations in β-tubulin isotype distribution, overexpression of MDR-1 or other drug transporters, specific changes in various components of signal transduction pathways (e.g. HER-2 overexpression) such as dysfunctional apoptosis regulating genes, and alterations in levels of endogenous regulators of microtubule dynamics, such as stathmin. These factors have motivated a search for novel antimitotic agents which share the same mechanism of action as paclitaxel but in other ways bypass these resistance mechanisms.

Of these proposed mechanisms of resistance, much work in the past has concentrated on investigating altered microtubule dynamics. Cabral, et al., have described a model in which resistance to tubulin-binding agents results from alterations in microtubule stability. (34,35). It remains unclear whether differential expression of specific isotypes of tubulin confer an altered paclitaxel response. However, several studies suggest that alterations in β-tubulin may confer paclitaxel resistance (28,35-42).

1. *In vitro* studies have demonstrated that β-tubulin subunit composition can alter the growing and shortening dynamics of microtubules and low levels of paclitaxel may alter these dynamics (21-27)
2. Class III β-tubulin depleted microtubules display increased sensitivity to paclitaxel-induced polymerization in vitro compared with unfractionated tubulin (36)
3. The paclitaxel-resistant murine cell fine J774.2 has increased expression of the class II β-tubulin isotype, Mβ2 (37)
4. A549 lung cancer cells selected for paclitaxel resistance display an altered β-tubulin isotype distribution compared with the parental non resistant line (38). In the same study, comparing untreated ovarian tumors from patients and paclitaxel-resistant malignant ascites, significant increases in mRNA of classes I (3.6 fold), III (4.4 fold), and IVa (7.6 fold) isotypes in the paclitaxel-resistant samples were observed (39)
5. Sickic et al have demonstrated that the KPTA5 cell line, which is intrinsically resistant to taxanes, displays increased expression of the class IVa tubulin isotype (40)
6. Complementing previous studies, others have described mutant β-tubulin in paclitaxel-resistant cell lines that exhibit impaired paclitaxel-driven polymerization (41‑43). A more recent report corroborates these findings in paclitaxel-resistant CHO cells that have revealed mutations affecting the Leucine cluster: Leu-215, -217,and -228. Using tet-regulated plasmid constructs, these mutations were introduced into a hemagglutinin antigen-tagged β-tubulin cDNA and transfected into wild-type Chinese hamster ovary cells. Low or moderate expression of the mutant gene conferred paclitaxel resistance; higher levels resulted in microtubule disassembly and cell cycle arrest at mitosis (43)
7. Finally, β-tubulin mutations located primarily in exon 4 of class IB tubulin, have been shown to correlate with response to paclitaxel in non-small cell lung cancer patients. Patients harboring mutations in β-tubulin had median survivals of 3 months compared with median survival of 10 months for patients without β-tubulin mutations(44).

The epothilones are a new class of more water-soluble non-taxane microtubule-binding agents obtained from the fermentation of the myxobacteria, Sorangium cellulosum. The chief components of the fermentation process are epothilones A and B. In 1994, the National Cancer Institute discovered that the epothilones possess potent cytotoxic activity. The cytotoxic activities of the epothilones, like those of the taxanes, have been linked to hyperstabilization of microtubules which results in mitotic arrest leading to cell death (32,33). Their chemical structure is distinct from that of paclitaxel. The epothilones are competitive inhibitors of the binding of [^3^H] paclitaxel to the microtubule (47), implying that they share the same or an overlapping binding site on the microtubule. Moreover, the epothilones are more potent than paclitaxel in various cell lines and retain their activity in paclitaxel-resistant cell lines that overexpress p-glycoprotein and in one cell line with acquired β-tubulin mutations (53). BMS-247550 is a semi-synthetic analog of the natural product epothilone B, specifically designed to overcome the metabolic instability of the natural product. Like paclitaxel, BMS-247550 blocks cells in: the mitotic phase of the cell division cycle and is a highly potent cytotoxic agent capable of killing cells at low nanomolar concentrations. Most importantly, BMS-247550 has demonstrated impressive antitumor activity in a number of preclinical human tumor models, including cancer cells that are inherently resistant to paclitaxel. These data demonstrate that BMS-247550 has the potential to be more efficacious than the current taxanes (49).

**2.2.1 Preclinical Antitumor Activity**

The following is a summary of the preclinical pharmacology of BMS 247550. More detailed information may be found in the Investigator Brochure (49).

In Vitro Assays

BMS 247550 has a broad spectrum of activity against a panel of tumor cell lines *in vitro*. In 18 of 21 cell lines tested, the concentration of BMS 247550 required to inhibit cell growth by 50% (IC_50_) was between 1.4-6nM. The cytotoxic activities of the epothilones are believed to be due to microtubule stabilization which results in mitotic arrest at the G2/M transition. In this regard, the potency of BMS 247550 is similar to those of its two natural analogs (Epothilone A and B) and comparable to paclitaxel. The concentration of BMS 247550 needed to arrest cells in mitosis corresponds well to the concentration required to kill cells over the same treatment duration. At a concentration close to the IC_90_  value (~7.5nM), BMS 247550 almost completely blocks cells in mitosis in 8 hours.

BMS 247550 is capable of substantially overcoming the resistance inherent in tumor cell lines known to be highly resistant to paclitaxel. HCT116/VM46 is a colon carcinoma cell line highly resistant to MDR agents because of greatly increased expression of the P-glycoprotein (Pgp) drug efflux pump. HCT116/VM46 is 155-fold more resistant to paclitaxel than the parent cell line HCT116, based on the IC_50_s. The resistance ratio is only 9.4 for BMS 247550. A2780Tax is resistant to paclitaxel because of a mutation in the tubulin protein. However, A2780Tax is only 1.9-fold more resistant to BMS 247550 than the parent cell line.

In Vivo Studies

BMS 247550 was evaluated *in vivo* in a panel of human and rodent tumor models, the majority of which were chosen because of their known, well-characterized resistance to paclitaxel. Paclitaxel sensitive models were included in order to gain a full assessment of the antitumor activity of BMS 247550. Significant, broad spectrum antitumor activity was demonstrated in most models tested by the parenteral route of administration. In selected models where both the parenteral and oral routes of administration were evaluated, BMS 247550 demonstrated comparable activity by either route.

In the Pat-7 clinically-derived paclitaxel-resistant ovarian model, BMS 247550 was administered intravenously (IV) to nude mice bearing staged tumors using an every 2 days x 5 schedule. At the optimal dose (4.8-6.3mg/kg/inj), BMS 247550 was highly active, eliciting 2.1 and 4.5 log cell kill (LCK) in two separate tests. Concomitantly evaluated IV paclitaxel yielded 0.6 and 1.3 LCKs, respectively, at its optimal dose.

A2780 is a fast-growing human ovarian carcinoma model that is highly sensitive to paclitaxel. Nude mice bearing staged tumors were treated with BMS 247550 using IV administration every 2 days x 5. At the maximum tolerated dose of 6.3mg/kg/inj, BMS 247550 was highly active yielding >4.8, 2 and 3.1 LCKs in three separate experiments. Concomitantly tested IV paclitaxel at its optimal dose, included in the first two studies, yielded 2 and 3.5 LCKs, respectively.

HCT116/VM46 is an MDR-resistant colon carcinoma. *In vivo*, grown in nude mice, HCT116/VM46 has consistently demonstrated high resistance to paclitaxel. In 12 consecutive studies, paclitaxel at its MTD elicited low LCKs ranging from 0-0.9 (median = 0.35 LCK). BMS 247550 treatment of mice bearing staged HCT116/VM46 tumors using the every 2 days x 5 IV administration schedule produced significant antitumor effects. At its optimal dose (4.8-6.3mg/kg/inj) in three separate studies, BMS 247550 yielded 3.1, 1.3, and 1.8 LCKs. In contrast, concomitantly tested IV paclitaxel yielded 0.4 and 0.7 LCKs, respectively, at MTD in the first two tests.

**2.2.2 Animal Toxicology**

The single-dose rat STD_10_ was determined to be 12.3mg/kg (74mg/m^2^). Peripheral neuropathy, bone marrow/lymphoid depression, and gastrointestinal and testicular changes were prominent; this was expected based on the common mechanism of action of BMS 247550 and paclitaxel. In mice, the severity of the BMS 247550 induced peripheral neuropathy at the maximum tolerated dose was similar to paclitaxel-induced peripheral neuropathy. In dogs, BMS 247550 produced severe toxicity and death at 100mg/m^2^, a dose higher than the rat single-dose STD_10_ of 74mg/m^2^. The deaths in the dogs receiving 100mg/m^2^ were attributed to severe gastrointestinal toxicity. A dose of 10mg/m^2^ (equivalent to 1/7 the rat STD_10_) was associated with transient minimal leukopenia and/or thrombocytopenia in one male and one female dog. The results of this study indicate that 1/10 of the rat STD_10_ (7.4mg/m^2^) would be well tolerated in dogs and should be a safe starting dose for the first-in-man Phase I trial. More detailed concerning animal toxicology may be found in the Investigator Brochure (49).

- - 1. **Pharmacokinetics**

Preclinical pharmacokinetics

Pharmacokinetic (PK) studies were conducted in mice, rats, and dogs after intravenous (IV) administration of BMS 247550. The following is a summary of those studies; more detailed information may be found in the Investigator Brochure (49).

Following single IV doses of 10, 25, and 30mg/kg, the mean Cmax values of BMS 247550 were in the range of 6422 to 24414ng/ml in male rats and 8384 to 25054ng/ml in female rats; AUC value ranged from 3864 to 19269h.ng/ml (males) and from 8156 to 34563h.ng/ml (female) (21). After single IV doses of 0.5 and 5mg/kg to dogs, the mean Cmax values for BMS 247550, combined across gender, were 218 and 5118ng/ml, respectively, and the mean AUC values were 316 and 6925h.ng/ml, respectively. In both species, dose-related increase in the systemic exposure (Cmax and AUC) of BMS 247550 was observed; however, the increase was more than proportional to the increase in dose. The AUC values of BMS 247550 and BMS-326412 were higher by 1.8- to 2.4-fold and 1.3- to 2.0-fold, respectively, in female rats compared to male rats. Gender effect on the kinetics of BMS 247550 could not be conclusively evaluated in the dog due to limited sample size, but the kinetics appeared to be reasonably similar between gender.

Multiple-dose intravenous toxicokinetic studies in rats and dogs were conducted. Results are summarized in the Investigator Brochure (49).

Following intravascular administration of BMS 247550 in mice (5mg/kg), rats (2mg/kg), and dogs (0.5mg/kg), the mean VSS values were 6.3, 23, and 25.2 l/kg, respectively. Comparison of these VSS values to the total body water of about 0.6-0.7 l/kg in mice, and rats, and dogs, respectively, suggests that BMS 247550 undergoes extensive extravascular distribution in these species.

BMS 247550 undergoes oxidation metabolism when incubated with mouse, rat, dog, and human liver microsomes. The rate of oxidative metabolism and the metabolite distribution appeared to be similar among these species. Qualitatively, there appeared to be similar production of metabolites of BMS 247550 after incubation with rat or human hepatocytes compared to microsomal incubations; however, products similar to those arising from the chemical degradation of BMS 247550 appeared to be the major products in the hepatocyte incubations. *In vitro*, BMS 247550 was a weak inhibitor of human CYP3A4 [average IC_50_ value of 7.3mcM (3.7mcg/ml)], but did not inhibit human CYP1A2, CYP2C9, CYP2C19, and CYP2D6, suggesting that BMS 247550 may have a minimal potential to alter the metabolic clearance of drugs that are highly metabolized by CYP3A4. When BMS 247550 was incubated with human liver microsomes along with compounds specific for the inhibition of individual cytochrome P450s, significant inhibition was observed only with the CYP3A4 inhibitors (troleandomycin and ketoconazole), suggesting that BMS 247550 may be a substrate for CYP3A4 in humans.

Following intravascular administration of BMS 247550 in mice (5mg/kg), rats (2mg/kg), and dogs (0.5mg/kg), the mean t½ values were approximately 3, 9.6, and 24h, respectively. CLT values were 68, 56, and 17.3ml/min/kg in mice, rats, and dogs, respectively; these values represented 76%, 100%, and 56% of the liver blood flow, respectively. In bile duct cannulated rats that received an intraarterial (10mg/kg) or oral (20mg/kg) dose of BMS 247550, negligible (<1% of the dose) excretion of intact BMS 247550 was observed in the bile, and some detectable amount (not quantified due to lack of stability data) of BMS 247550 was also observed in the urine.

Clinical Pharmacokinetics

Preliminary pharmacokinetic data are available from the on-going first-in-human study (CA163-001) conducted with BMS 247550. This study is an open-label, Phase II study in melanoma patients. Each treatment cycle consists of three weekly doses of BMS 247550 and each cycle is repeated every 28 days. Plasma pharmacokinetics are being assessed on Cycle 1 and 2. Pharmacokinetic data are available from the following dose levels: 7.4, 15, 30, 50, 57, and 65 and are presented in the table below:

| Parameter  (Units) | 7.4  mg/m^2^ | 15  mg/m^2^ | 30  mg/m^2^ | 50  mg/m^2^ | 57  mg/m^2^ | 65  mg/m^2^ |
| --- | --- | --- | --- | --- | --- | --- |
| Cycle 1 | | | | | | |
| Sample size | n=3 | n=3 | n=3 | n=6 | n=2 ^a^ | n=1 ^a^ |
| Cmax  (ng/mL) | 199  (97) | 195  (124) | 495  (398) | 841  (360) | 1056 | 1056 |
| AUC(INF)  (h.ng/mL) | 537  (52) | 844  (342) | 1364  (432) | 3076  (1432) | 3947 | 2526 |
| T-HALF  (h) | 29.5  (19.3) | 48.1  (10.1) | 37.0  (7.2) | 32.8  (11.1) | 34.6 | 28.7 |
| MRT(INF)  (h) | 29.9  (21.0) | 56.4  (20.1) | 36.2  (7.4) | 32.1  (10.3) | 24.1 | 20.6 |
| CLT  (mL/min/m^2^) | 230  (20.7) | 327  (114) | 398  (146) | 318  (127) | 252 | 429 |
| VSS  (L/m^2^) | 399  (252) | 1157  (736) | 901  (498) | 554  (115) | 360 | 530 |
| Cycle 2 | | | | | | |
| Sample size | n=1 ^a^ | n=3 | n=3 | n=2 ^a^ |  |  |
| Cmax  (ng/mL) | 178 | 318  (148) | 494  (193) | 597 |  |  |
| AUC(INF)  (h.ng/mL) | 372 | 1261  (722) | 1592  (539) | 2251 |  |  |
| T-HALF  (h) | 16.9 | 34.5  (3.3) | 43.5  (21.0) | 31.4 |  |  |
| MRT(INF)  (h) | 15.0 | 37.3  (5.2) | 46.1  (27.9) | 31.6 |  |  |
| CLT  (mL/min/m^2^) | 332 | 243  (119) | 341  (105) | 399 |  |  |
| VSS  (L/m^2^) | 298 | 536  (266) | 828  (316) | 708 |  |  |

a Standard deviation not presented when N < 3.

Across doses of 7.4 to 57 mg/m^2^  (65 mg/m^2^  not included since n=1), mean AUC values of BMS 247550 for cycle 1 increased in the ratio of 1:1.6:2.5:5.7:7.4 for doses in the ratio of 1:2:4:6.7:7.7. Mean CLT values of BMS 247550 for Cycle 1 ranged from 230 to 398 mL/min/m^2^; these values represents 30% to 51% of the average hepatic blood flow (780 mL/min/m^2^). Mean VSS values for cycle 1 across doses were in the range of 399 to 1157 L/m^2^ and is suggestive of extensive extravascular distribution. The PK parameters of BMS 247550 appeared to be reasonably similar across Cycles 1 and 2.

Comparison of Plasma Exposures in Humans to Plasma Exposures Observed In Preclinical Toxicokinetic Studies:

Comparison of the human plasma exposure data (Cmax and AUC) from the ongoing first-in-human study (CA163-001) at the 50 mg/m^2^ dose level with the plasma exposures observed in single dose IV rat and dog toxicokinetic studies (51,52) is provided in the table below:

| Species | IV Dose  (mg/m^2^) | Cmax (ng/mL)^a^  [multiple of human exposure] | AUC(INF) (h.ng/mL)  [multiple of human exposure] |
| --- | --- | --- | --- |
| Rat  Male (N = 3)  Female (N = 3) | 60  60 | 6422 [7.6]  8384 [10.0] | 3864 [1.3]  8156 [2.7] |
| Dog (2M, 2F)^b^ | 10 | 218 [0.3] | 258 [0.1] |
| Dog (2M, 2F)^b^ | 100 | 5118 [6.1] | 6925 [2.3] |
| Human (N = 6) | 50 | 841 [1.0] | 3076 [1.0] |

^a^ Dosing in animals was a 10-minute infusion while that in humans was a 1-h infusion.

^b^ Combined across gender due to small sample size N = 2/gender.

AUC values of BMS 247550 at the lowest dose tested in rat toxicokinetic studies (60mg/m^2^, close to the STD_10_ of 74mg/m^2^) were higher by 1.33 and 2.9-fold in male and female rats, respectively, compared to the exposures in humans at a dose of 50mg/m^2^. At the well-tolerated dose of 10mg/m^2^ in dogs, the plasma AUC was about 11.9-fold lower as compared to the exposure in humans at 50mg/m^2^. However, at the lethal dose of 100mg/m^2^ in dogs, the plasma AUC values in dogs were higher by 2.3-fold compared to the plasma AUC observed in humans at 50mg/m^2^.

Metabolism in Humans and Potential for Metabolic Drug Interactions

Formal characterization of BMS 247550 metabolism in humans has not been completed. BMS 247550 undergoes oxidative metabolism when incubated with human liver microsomes *in vitro*. When BMS 247550 was incubated with human liver microsomes along with compounds specific for the inhibition of individual cytochrome P450 enzymes, almost complete inhibition was observed only with the CYP3A4 inhibitors (troleandomycin and ketoconazole) suggesting that BMS 247550 may be a substrate for CYP3A4 in humans.

Formal drug interactions studies of BMS 247550 have not been completed. BMS 247550 did not inhibit human CYP1A2, CYP2C9, CYP2C19, or CYP2D6 *in vitro* at concentrations up to 200mcM. BMS 247550 was a weak inhibitor of human CYP3A4 *in vitro* [average IC_50_ value of 7.3mcM (3.7mcg/ml)]. Given that BMS 247550 is being administered on an intermittent schedule (q21 days) and is a weak inhibitor of CYP3A4 *in vitro* and plasma concentrations are generally less than the IC50 for CYP3A4 inhibition *in vitro*, the likelihood for clinically important pharmacokinetic drug interactions with concomitantly administered medications which are dependent on CYP3A4 metabolism is limited.

Preliminary Studies

Defining a structure activity relationship (SAR) for the epothilones: In collaboration with Dr. Samuel Danishefsky from the Sloan-Kettering Institute for Cancer Research and the Department of Chemistry at Columbia University, the Horwitz laboratory was the first to report a SAR for the epothilones (55). A large number of epothilone analogs, derived from epothilone A, epothilone B and desoxyepothilone, have been evaluated. The epothilones are defined by three arbitrary sectors: aryl (1: CI-C8), alkyl (16), and acyl (17). The aryl sector is very sensitive to modifications, whereas the alkyl and acyl sectors are more tolerant (Figure 1). It has also been determined that the potency of epothilone B exceeds that of epothilone A.


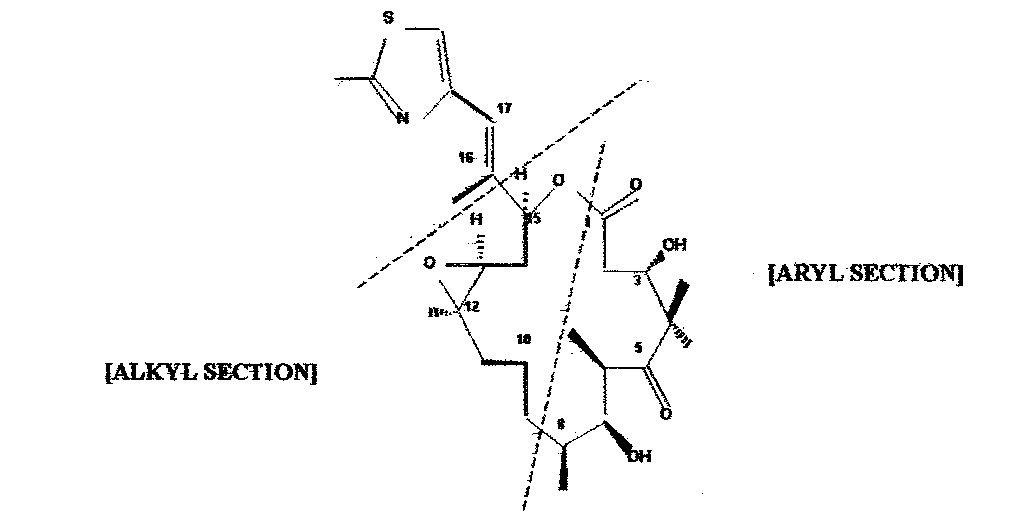


Figure 1: Chemical structure

The significance of β-tubulin isotype distribution and mutations to taxane resistance and clinical response: Alterations in β-tubulin isotype distribution in paclitaxel-resistant cell lines (37,39) and in untreated and paclitaxel-resistant ovarian cancer cells (36) have been demonstrated and these may be associated with resistance to paclitaxel. Changes in expression profiles of six β-tubulin isotypes were analyzed using RT-PCR analysis of cDNA extracts prepared from cell lines and tumors. In paclitaxel- and docetaxel-resistant murine cell lines, increased expression of both α-and β-tubulin (1.6- 2.9 fold) was observed in all cell lines tested by Western blotting. In one of these cell lines, which overexpresses MDR-1, the class II isotype of β-tubulin was upregulated 21-fold, although its expression was barely detectable in the parental cell line (37). This study led to an evaluation of a paclitaxel-resistant human lung cancer cell line and human tumor cells that do not overexpress p-glycoprotein (39). The paclitaxel-resistant cell line displayed an altered ratio of classes I, II, III and IVa β-tubulin isotypes, while the paclitaxel-resistant tumor samples exhibited increases in class I, III and IVa isotypes, compared to primary untreated tumors. Subsequently, antisense oligonucleotides were designed to the class III β-tubulin isotype, and examined for their efficacy in reducing mRNA and protein expression (36). A concentration-dependent reduction in class III isotypes by antisense was associated with a decrease in class III protein expression, which corresponded to a 39% increase in sensitivity to paclitaxel, suggesting a potential role for this isotype in paclitaxel resistance. The relevance of these findings to human tumors requires clarification, and we will address this in our studies with BMS-247550 pre- and post-treatment tumor samples. These data suggest that the isotype composition of microtubules may influence sensitivity to tubulin-binding agents such as BMS-247550. However, the tubulin isotype profile of mammalian cells is complex and variable from one tissue to another. At present, no simple relationship has been proposed between the level of expression of a given isotype and the degree of sensitivity or resistance to paclitaxel.

Mutations in Class I β-tubulin (the predominant isoform in most cell types) have been described in paclitaxel-resistant cancer cell lines (43). In one of these studies, mutations in the Class I isotype of the β-tubulin are localized to residues 270 and 364 in two separate paclitaxel-resistant cell lines that do not overexpress P-glycoprotein. The Horwitz laboratory have also isolated a paciltaxel-resistant variant of MDA-MB-231 breast cancer cells that do not overexpress P-glycoprotein, but which have a mutation in the Class I β-tubulin at amino acid position 198 (glu→gly) (38), in addition to several epothilone-resistant cells lines and one cell line that is resistant to BMS-247550, all of which have mutations at various locations in Class I β-tubulin. An analysis of Class I β-tubulin in nine paclitaxel-resistant Chinese hamster ovary (CHO) cell lines revealed a cluster of mutations, six of which result in an amino acid substitution of leucine at codon 215 (44), a highly conserved position in β-tubulin, adjacent to the domains this laboratory previously identified as binding to paclitaxel using photoaffinity analogs (20). Importantly, this is the first study to demonstrate that overexpression of this mutation in normal CHO cells confers resistance to paclitaxel, together with reduced β-tubulin acetylation, suggesting that this mutant form of tubulin destabilizes microtubules. Additionally, A549 lung cancer cells and their epothilone B resistant variants also do not express P-glycoprotein. These cells have a mutation at amino acid 292 (glu →glutamic acid), in a region that is within the proposed taxane binding domain.

**2.2.4 Phase I Studies**

The Phase I program with BMS 247550 has tested two different dose escalation schedules of administration: *CA163-001*: Day 1 of a 21-day cycle (q3 weeks) and *CA163-002*: Day 1, 8, 15 of a 28-day cycle (q week). The weekly schedule was chosen for this Phase II study based on evidence of profound neurotoxicity at the every 3 week schedule. (Correspondence from NCI to Epothilone Principal Investigators following NCI Phase I meeting 10/01) and results of the Phase I trial (CA 163-002).

##### Study CA163-001

Thirty-one patients have been enrolled in the CA163-001 study at 7.5, 15, 30, 50, 57, and 65 mg/m^2^. Enrollment was completed in August 2000 and 16 patients continue to receive subsequent cycles of BMS 247550 at doses of 40 (1 patient dose reduced), 50 (14 patients) and 57 (1 patient) mg/m^2^. Dose limiting toxicities observed at 65 mg/m^2^ included neuropathy, myalgia, arthralgia and neutropenia. One case of pneumonia leading to fatal pneumococcal sepsis occurred at the 50 mg/m^2^ dose level. Other observed toxicities (n=14) considered related to BMS 247550 were fatigue (43%), myalgia (29%), arthralgia (21%), alopecia (21%), nausea (21%), constipation (14%), vomiting (14%), and sensory neuropathy (14%). In addition to tingling and numbness, the neuropathy has been described to include pain in the extremities in some patients. Confirmed objective responses have been observed in one patient with melanoma and in taxane-pretreated patients, including one each with non-small cell lung cancer, with peritoneal (ovarian) cancer and with adenocarcinoma of unknown primary. After accrual to the dose escalation phase of the trial, 10 additional patients were accrued at a dose of 50 mg/m^2^. One patient in this group has required dose reduction for febrile neutropenia. Based on this Phase I experience, 50 mg/m^2^ given every three weeks has been selected as the Phase II dose. Routine premedication was started in all new patients after one occurrence of a hypersensitivity reaction (HSR) in this study at the 30 mg/m^2^ dose level; since introduction of premedication, no further significant hypersensitivity reactions occurred.

##### Study CA163-002

In an ongoing Phase I study (CA163-002, Dr. Martine Piccart, Institut Jules Bordet, Brussels, Belgium), which started end of December 1999, BMS 247550 is given as a continuous weekly intravenous 30 minute infusion to patients with solid tumors. As of mid-July 2000 all new patients in this study also receive a single oral dose (cycle -1) of BMS 247550 on Day -6 before cycle 1. Plasma sampling is performed both during cycle -1 and cycle 1 to evaluate the pharmacokinetics and pharmacodynamics of BMS 247550 and the absolute oral bioavailability of BMS 247550. Routine premedication was started in all new patients after the second occurrence of a hypersensitivity reaction (HSR) in this study.

As of the end of August 2000, 17 patients have been treated on this dose-finding study. Three patients have been entered at the current dose level of 20 mg/m^2^. Together they have received 12 weekly administrations of BMS 247550 (3 of which were given orally). There have been no DLTs observed so far and after the introduction of standard premedication, no further HSRs occurred. The drug related toxicities reported so far are mild to moderate myalgia/arthralgia, asthenia, diarrhea, sensory neurotoxicity, anorexia and alopecia. One patient experienced hypotension CTC Grade 1 and bradycardia CTC Grade 2 after the 5th infusion of 10 mg/m² intravenous BMS 247550. These events were possibly related to the drug.

**2.2.5 Potential For Hypersensitivity Reactions Related To Polyoxyethylated Castor Oil (Cremophor®EL)**

Based on the presence of polyoxyethylated castor oil in the formulation of BMS 247550, the potential for hypersensitivity reactions exists with intravenous administration of this compound. In clinical trials of paclitaxel, anaphylaxis and severe hypersensitivity reactions (dyspnea requiring bronchodilators, hypotension requiring treatment, angioedema, and generalized urticaria) have occurred in 2% of patients (56). Three of the first twenty patients treated with BMS 247550 have experienced a hypersensitivity reaction. Since instituting routine premedication, none of the remaining patients have experienced a significant hypersensitivity reaction. Hence, routine premedication will be used in this trial (see Section 4.1.1 for prophylaxis for hypersensitivity reactions).

**2.3 Rationale**

The Phase I program with BMS 247550 has tested two different dose escalation schedules of administration: Day 1 of a 21-day cycle (q3 weeks) and Day 1, 8, 15 of a 28-day cycle (q week). The weekly schedule was chosen for this Phase II study based on evidence of profound neurotoxicity at the every 3 week schedule. (Correspondence from NCI to Epothilone Principal Investigators following NCI Phase I meeting 10/01) and results of the Phase I trial (CA 163-002).

**3. PATIENT SELECTION**

**3.1 Eligibility Criteria**

3.1.1 Patients must have histologically or cytologically confirmed malignant melanoma stage IV.

3.1.2 Patients must have measurable disease, defined as at least one lesion that can be accurately measured in at least one dimension (longest diameter to be recorded) as >20 mm with conventional techniques or as >10 mm with spiral CT scan. See section 9.1.1 for the evaluation of measurable disease.

3.1.3 Prior therapy

Two subgroups of patients will be treated:

**Subgroup A: Chemotherapy naïve**

These patients may not have received any prior chemotherapy

**Subgroup B: DTIC pretreated**

These patients may have received a maximum of two prior lines of chemotherapy, and must have received DTIC or temozolomide.

Prior vaccine therapy, immunotherapy (e.g. IL-2, interferon) and radiotherapy are permitted in both subgroups. Patients treated with limb-perfusion are eligible and will be assigned to Subgroup B.

No minimum period since last therapy is defined, but patients must have adequate organ toxicity as defined in 3.1.7, and preexisting non-hematological dysfunction <grade 2.

3.1.4 Age ≥18 years.

Because no dosing or adverse event data are currently available on the use BMS 247550 in patients <18 years of age, children are excluded from this study but will be eligible for future pediatric single-agent trials, if applicable.

3.1.5 Life expectancy ≥3 months.

3.1.6 ECOG performance status 0-2 / Karnofsky ≥60% (see Appendix B).

3.1.7 Patients must have normal organ and marrow function as defined below:

- absolute neutrophil count ≥1.5 x 10^9^/l
- platelets ≥100 x 10^9^/l
- total bilirubin within normal institutional limits
- AST (SGOT) / ALT (SGPT) ≤2.5 x upper limit of normal
- creatinine ≤1.5 x upper limit of normal

3.1.8 Patients with known brain metastases may be included in this study if they fulfill all of the following criteria:

- the lesions have remained radiologically stable for at least six weeks after completion whole brain irradiation, and remain stable at the time of study entry
- there is no mass effect present radiologically
- the patient is no longer receiving steroids to control symptoms of brain metastases

3.1.9 The effects of BMS 247550 on the developing human fetus at the recommended therapeutic dose are unknown. Women of child-bearing potential and men must therefore agree to use adequate contraception (hormonal or barrier method of birth control) prior to study entry and for the duration of study participation. Should a woman become pregnant or suspect she is pregnant while participating in this study, she should inform her treating physician immediately.

3.1.10 Ability to understand and the willingness to sign a written informed consent document.

**3.2 Exclusion Criteria**

3.2.1 Patients may not be receiving any other investigational agents.

3.2.2 History of severe allergic reactions (Grade III or IV or Grade II not responsive to steroids) attributed to taxanes or medications containing Cremophor®EL.

3.2.3 Patients with preexisting peripheral neuropathy of grade 2-4 are excluded from this study

3.2.4 Uncontrolled intercurrent illness including, but not limited to, ongoing or active infection, symptomatic congestive heart failure, unstable angina pectoris, cardiac arrhythmia, or psychiatric illness that would limit compliance with study requirements.

3.2.5 Pregnant women are excluded from this study because the teratogenic or abortifacient effects of BMS 247550 are unknown. Because there is an unknown but potential risk for adverse events in nursing infants secondary to treatment of the mother with BMS 247550, breastfeeding should be discontinued if the mother is treated with BMS 247550.

- - 1. Because patients with immune deficiency are at increased risk of lethal infections when treated with marrow-suppressive therapy, HIV-positive patients receiving combination anti-retroviral therapy are excluded from the study because of possible pharmacokinetic interactions with BMS 247550. Appropriate studies will be undertaken in patients receiving combination anti-retroviral therapy when indicated.

**3.3 Inclusion of Women and Minorities**

*Both men and women and members of all ethnic groups are eligible for this trial, although accrual of non-white patients is expected to be low as melanoma is rare in non-white ethnic groups. Based on accrual in previous melanoma studies at NYU and Sydney, the proposed study population is illustrated in the table below:*

|  | **Race/Ethnicity** | | | | | |
| --- | --- | --- | --- | --- | --- | --- |
| **Gender** | White, not of Hispanic Origin | Black, not of Hispanic Origin | Hispanic | Asian or Pacific Islander | Unknown | Total |
| Male | 60% | 0 | 0 | 0 | 0 | 60% |
| Female | 40% | 0 | 0 | 0 | 0 | 40% |
| Total | 100% | 0 | 0 | 0 | 0 | 100% |

**4. TREATMENT PLAN**

**4.1 Agent Administration**

Treatment will be administered on an outpatient basis. Expected adverse events and appropriate dose modifications for BMS 247550 are described in Section 5. No investigational or commercial agents or therapies other than those described below may be administered with the intent to treat the patient’s malignancy. **Note**: St. John’s Wart significantly induces cytochrome p450 3A4 and may affect the metabolism of BMS 247550. Administration is not permitted on this study.

###### 4.1.1 Premedication

Since BMS 247550 is formulated in polyoxyethylated castor oil (Cremophor®EL), hypersensitivity reactions may occur. Therefore, patients should be monitored closely for any signs or symptoms of hypersensitivity and emergency equipment must be available to treat possible anaphylactic/ anaphylactoid reactions as outlined in Section 4.2.2.

**All patients will receive the following premedication to minimize nausea, vomiting and hypersensitivity reactions:**

**Diphenhydramine** 50mg IV (or equivalent,) 1 hour prior to BMS 247550

**Ranitidine** 50mg IV **(or equivalent)**, 1 hour prior to BMS 247550

**Ondansetron** 8mg IV/oral **(or equivalent)**, immediately prior to BMS 247550

**Dexamethasone** 20mg IV, immediately prior to BMS 247550

###### 4.1.2 BMS 247550

All patients will receive:

**BMS 247550 20 mg/m2, 1 hour infusion, weekly x3 followed by 1 week break**

This dose may subsequently be reduced for individual patients, depending on toxicity (Section 5.2). In calculating surface areas, actual heights and weights should be used; that is, there should be no adjustment to “ideal” weight. The total dose delivered should be rounded to the nearest mg.

See Section 6.1.5 for details on preparation of BMS 247550 for intravenous administration.

An infusion pump must be used to deliver BMS 247550 over one hour during cycle 1 in which pharmacokinetics is collected. After confirming that the required volume has been administered, solution remaining in the line and container should be disposed of per institution policies for cytotoxic disposal. The patient’s blood pressure and heart rate should be measured at baseline and every 15 minutes during the infusion of BMS 247550, and a nurse must be present in the immediate treatment area throughout the infusion. A physician must be in close proximity to the patient treatment area.

**4.2 Supportive Care Guidelines**

**4.2.1 Growth Factors**

Hematological growth factors will not be used prophylactically in this study, but may be used at the discretion of the investigator in the event of severe hematological toxicity.

##### 4.2.2 Hypersensitivity Reactions

**Treatment**

In case of hypersensitivity reactions, the Investigator should institute treatment measures deemed medically appropriate. Based on prior experience with paclitaxel, the following treatment recommendations may be applicable:

CTC Grade 1 Allergic Reaction/Hypersensitivity (transient rash, drug fever <38°C):

- Supervise at the bedside

CTC Grade 2 Allergic Reaction/Hypersensitivity (urticaria, drug fever ≥38°C, and/or asymptomatic bronchospasm):

- Interrupt the infusion of BMS 247550
- Give IV antihistamines (diphenhydramine 25-50mg, and ranitidine or famotidine)

**Note**: Cimetidine inhibits cytochrome p450 3A4 and may effect the metabolism of BMS 247550, resulting in increased blood drug concentrations. This H2 blocker is therefore not permitted on this trial.

- After recovery of symptoms, resume the infusion at a slower rate and if no further symptoms appear, complete the administration of the dose. (Note: It is recommended that the infusion of BMS 247550 be completed within 3 hours of the final dilution. See Section 6.1.6).
- If symptoms recur, stop the infusion and proceed with interventions listed in CTC Grade 3 or 4 below.

CTC Grade 3 or 4 Allergic Reaction/Hypersensitivity (symptomatic bronchospasm requiring parenteral medication(s) with or without urticaria; allergy-related edema/angioedema; anaphylaxis):

- Stop the infusion of BMS 247550.
- Administer additional doses of H1 and H2 blockers intravenously.
- Administer epinephrine, and nebulized bronchodilators as medically indicated.
- Consider IV steroids which may prevent recurrent or ongoing reactions.
- If grade 4, report adverse event (see Section 10.2)
- Patient off study, or rechallenge with premedication (see below) after 24 hours, based upon the judgment of the Investigator and the Protocol Chair

**Rechallenge**

Suggested regimen for rechallenge of BMS 247550 after a Grade 3 or 4 hypersensitivity reaction despite recommended premedication:

- **Dexamethasone** 20 mg PO at 12 and 6 hours before BMS 247550 infusion.
- **Diphenhydramine** 50mg IV 30minutes before BMS 247550.
- **Ranitidine** 50mg or **Famotidine** 20mg IV 30 minutes before BMS 247550.
- Begin BMS 247550 at **25% of the previous rate** for 1 hour;
- **Increase rate gradually** to complete the total infusion within 3 hours from the time the drug was diluted in Lactated Ringer’s Injection (LRI) (see Section 7.2.1).

**4.2.3 Contraception**

Women of childbearing potential and sexually active males are strongly advised to use an effective method of contraception.

**4.2.4 Other**

Other appropriate supportive care medications may be administered at the investigator’s discretion.

**4.3 Duration of Therapy**

In the absence of treatment delays due to adverse event(s), treatment may continue until one of the following criteria applies:

- Disease progression,
- Intercurrent illness that prevents further administration of treatment,
- Unacceptable adverse event(s)
- Patient decides to withdraw from the study
- General or specific changes in the patient’s condition render the patient unacceptable for further treatment in the judgment of the investigator.

**5. EXPECTED ADVERSE EVENTS / DOSE MODIFICATIONS**

**5.1**  **Expected Adverse Events Associated with BMS 247550**

The following adverse events have been associated with BMS 247550:

Common (>20% patients)

- Leukopenia / neutropenia (dose limiting)
- Thrombocytopenia
- Anemia
- Fatigue
- Alopecia
- Myalgia
- Arthralgia
- Sensory neuropathy
- Constipation

**Occasional (5-20% patients)**

- Nausea
- Vomiting
- Mucositis
- Rash
- Anorexia
- Diarrhea
- Insomnia
- Febrile neutropenia

Rare (<5% patients)

- Edema
- Allergy / hypersensitivity
- Muscle weakness
- Radiation recall phenomenon
- Tumor pain flare
- Pulmonary infiltrates/pneumonitis
- Taste disturbance
- Death

**5.2 Dosing Delays / Dose Modifications**

Prior to retreatment, patients must have recovered the following organ function:

- absolute neutrophil count ≥1.0 x 10^9^/l
- platelets ≥100 x 10^9^/l
- total bilirubin within normal institutional limits
- AST (SGOT) / ALT (SGPT) ≤2.5 x upper limit of normal
- creatinine ≤1.5 x upper limit of normal
- other Grade 0-1

Any patient experiencing the relevant toxicities (cited below in section 5.2) after one dose reduction will be removed from the protocol.

Patients should be dose reduced to 16 mg/m^2^ IV (20% dose reduction) over 1 hour in the event of any of the following:

- grade 4 neutropenia ≥5 days
- febrile neutropenia
- grade 4 thrombocytopenia
- grade 3 non-hematological toxicity
- 1 week delay for recovery from any toxicity

No patients should have his / her dose re-escalated following dose reduction.

**6. AGENT FORMULATION AND PROCUREMENT**

**6.1 Study Agent BMS 247550 (NSC #710428)**

**Chemical Name:** 1S-[1R*,3R*(E),7R*,10S*,11R*,12R*,16S*]]-7,11-Dihydroxy-8,8,10,12,16-pentamethyl-3-[1-methyl-2-(2-methyl-4-thiazolyl)ethenyl]-17-oxa-4-azabicyclo[14.1.0]heptadecane-5,9-dione.

**Other Names:** Epothilone B analog

**Molecular Formula:** C_27_H_42_N_2_O_5_S **M.W.:** 506.7 grams/mole

**Mode of Action:** BMS-247550 is a semi-synthetic analog of the natural product epothilone B. The epothilones are a novel class of non-taxane microtubule-stablizing agents obtained from the fermentation of cellulose degrading myxobacteria, *Sorangium cellulosum.*

**Description:** Epothilone B analog

**How Supplied**: BMS-247550 appears as a lyophilized, white to off-white color, whole or fragmented cake in a vial. The drug product is available in three different potencies: 15 mg/vial, 20 mg/vial, and 30 mg/vial.

Vial(s) containing Vehicle for Constitution of BMS-247550 for Injection, 8 mL/vial, 5.5 mL/vial, or 16.5 mL/vial are supplied with the lyophilized drug product The Vehicle for Constitution of BMS-247550 for injection (diluent) appears clear to slightly hazy and is colorless to pale in color. The diluent is comprised of an ethanol plus polyoxyethylated castor oil (Cremophor® EL) mixture (1:1 by volume).

One vial of the 8 mL/vial vehicle product is provided with the 15 mg/vial of BMS-247550 for Injection. Two vials of the 5.5 mL/vial vehicle product are provided with the 20 mg/vial of BMS-247550 for Injection. One vial of the 16.5 ml/vial vehicle product is provided with the 30 mg/vial of BMS-247550 for Injection.

The drug vial should only be reconstituted with the provided diluent. The diluents are not interchangeable and should only be utilized to reconstitute a particular strength.

Preparation: Prior to constitution of the lyophile, the diluent vial (s) should be kept at room temperature for approximately one hour. Inject 8 mL, 11 mL, and 16.5 mL of the diluent into the 15 mg vial, 20 mg vial, and 30 mg vial, respectively. Gently swirl the vial until the lyophile is completely dissolved. In all cases, this results in a 2mg/mL solution. The vial sizes and the required amount of diluent are summarized in the following table:

| BMS-247550 vial size | Diluent provided | Volume of diluent needed for reconsitution of drug vial | Final  concentration |
| --- | --- | --- | --- |
| 15 mg | One 8 mL vial | 8 mL | 2 mg/mL |
| 20 mg | Two 5.5 mL vials | 11 mL | 2 mg/mL |
| 30 mg | One 16.5 mL vial | 16.5 mL | 2 mg/mL |

This solution must be further diluted with Lactated Ringer’s Injection (LRI) to a final BMS-247550 concentration ranging from 0.1 mg/mL to 0.6 mg/mL in a non-PVC container before administration to the patient. Any remaining solution should be discarded according to institutional procedures for cytotoxics.

*Note:* 15 mg/vial potency: The label fill for the drug is 15 mg/vial which is to be constituted to a concentration of 2 mg/mL with the diluent. To account for vial/needle/syringe (VNS) loss, the actual amount of drug in the vial is 16 mg (+ 3%). Hence, the drug should be constituted using 8 ml of the vehicle for constitution (to achieve a BMS-247550 concentration of 2 mg/mL).

*Note:* 20 mg/vial potency: The label fill for the drug is 20 mg/vial which is to be constituted to a concentration of 2 mg/mL with the diluent. To account for vial/needle/syringe (VNS) loss, the actual amount of drug in the vial is 22 mg (+ 3%). Hence, the drug should be constituted using 11 mL of the vehicle for constitution (to achieve a BMS 247550 concentration of 2 mg/mL).

*Note:* 30 mg/vial potency: The label fill for the drug is 30 mg/vial which is to be constituted to a concentration of 2 mg/ml with the vehicle. To account for vial/needle/syringe (VNS) loss, the actual amount of drug in the vial is 33 mg (+ 3%). Hence, the drug should be constituted using 16.5 mL of the vehicle for constitution (to achieve a BMS-247550 concentration of 2 mg/mL).

**Storage**: BMS-247550 for Injection and the diluent for constitution should be stored refrigerated (2° to 8°C) prior to use and protected from light.

**Stability:** Shelf life surveillance is ongoing. After initial constitution with the accompanying diluent, the product may be stored for a maximum of 24 hours at room temperature and room light. After final dilution in Lactated Ringers for Injection (LRI) to concentrations between 0.1 and 0.39 mg/mL, the drug product is stable at room temperature and light for a maximum of 3 hours. When diluted with Lactated Ringers for Injection (LRI) to concentrations between 0.4 and 0.6 mg/mL, the drug product is stable at room temperature and light for a maximum of 6 hours.

CAUTION: The single-use lyophilized dosage form contains no antibacterial preservatives. Therefore, it is advised that the reconstituted product be discarded 8 hours after initial entry.

Route of Administration: The BMS-247550 infusion must be administered intravenously through an appropriate in-line filter with a microporous membrane of 0.22 to 5.0 microns. (See Incompatibilities section for a list of appropriate filters.) Lactated Ringers Injection (LRI) should be used to flush the IV line or extension set at the end of the infusion, if flushing is required.

**Reported Adverse Events and Potential Risks:**

Allergic reactions (anaphylaxis)/hypersensitivity (including drug fever), leukopenia, neutropenia, granulocytopenia, sinus bradycardia, edema, hypotension, fatigue (lethargy, malaise, asthenia), fever, alopecia, dermatitis, flushing, pruritus, radiation recall reaction, rash/desquamation, anorexia, constipation, diarrhea, nausea, stomatitis, dysgeusia, vomiting, febrile neutropenia, infection, muscle weakness (not due to neuropathy), dizziness/lightheadedness, insomnia, motor neuropathy, tingling, numbness, speech impairment (dysphasia or aphasia), syncope, arthralgia, myalgia, neuropathic pain, phantom limb pain, post-infection neuralgia, painful neuropathies, pain in extremities, cough, dyspnea, hiccoughs, hypoxia, pneumonitis/pulmonary infiltrates, urinary retention.

Also reported on BMS-247550 trials but with the relationship to BMS-247550 still undetermined: decreased hemoglobin, thrombocytopenia, hyperglycemia, hyperkalemia, hypocalcemia, transaminitis, ataxia, cerebrovascular ischemia (1 case), seizures (1 case).

**Potential Drug Interactions:** BMS-247550 may have a minimal potential to alter the metabolic clearance of drugs that are

highly metabolized by CYP3A4. When BMS-247550 was incubated with human liver microsomes along with compounds

specific for the inhibition of individual cytochromeP450s, significant inhibition was observed only with the CYP3A4 inhibitors

(troleandomycin and ketoconazole) suggesting that BMS-247550 may be a substrate for CYP3A4 in humans.

**Incompatibilities:** Contact of the diluted product with polyvinyl chloride (PVC) equipment or devices that are plasticized

with di- (2-ethylhexyl)pthalate (DEHP) should be avoided to prevent leaching of DHEP into the infusion medium. In order

to minimize patient exposure to DEHP, which my be leached from PVC infusion bags or sets diluted BMS-247550

solutions should preferably be stored in bottles (glass, polypropylene) or plastic bags (polypropylene, polyolefin) and

administered through polyetheylene-lined administration sets or PVC sets plasticized with TOTM (trioctyl trimellitate). IV

sets and components typically used for the administration of paclitaxel have been found to be compatible with infusions of

BMS-247550.

The following infusion components have been qualified for use with BMS-247550**:**

- IV sets containing an in-line 0.22 micron filter
  1. Baxter Vented Paclitaxel Set (Catalog #2C7553)
  2. Abbott Primary IV Plumset (Catalog #11947)
- IV sets not containing an in-line 0.22 micron filter (need to add one of the filters below)
  1. McGaw AccuPro Pump Nitroglycerin IV Set (Catalog #V8333)
  2. Clintec IV Fat Emulsion Set (Catalog #2C1105)
- Filter extension set (to be used with IV sets not containing an in-line filter)
  1. Braun Filter Extension Set with 5 Micron Filter (Catalog #FE-5010Y)

**Patient Care Implications:**

Suggested Regimen for Treatment of Hypersensitivity Reactions

Primary prophylaxis (1 hour to 30 minutes prior to BMS247550):

- H1 blocker (diphenhydramine 50 mg po or IV)

H2 blocker (ranitidine 150 mg po or 50 mg IV or another equivalent H2 blocker)

Treatment and secondary prophylaxis:

- CTC Grade I Allergic/Hypersensitivity Reaction (transient rash, drug fever < 38°C): Supervise without further treatment.
- CTC Grade 2 Allergic/Hypersensitivity Reaction (urticaria, drug fever greater than or equal to 38°C and/or asymptomatic bronchospasm): Interrupt the infusion. If symptoms abate, attempt re-infusion at a slower rate. If the symptoms recur, discontinue infusion and follow guidelines below.
- Recurrent CTC Grade 2 or CTC Grade 3 or 4 Allergic/Hypersensitivity Reactions: Stop the infusion. Administer additional doses of H1 and H2 blockers intravenously. Administer IV steroids (see discussion below) and consider epinephrine and bronchlodilators as clinically indicated. Further treatment should be delayed 24 hours. Prior to rechallenge and with all subsequent cycles, give both an H1 and H2 blocker intravenously plus dexamethasone 20 mg x 2 doses (orally or intravenously) 12 and 6 hours pre BMS247550. Dexamethasone could be used at the investigator’s discretion in subsequent cycles for recurrent (i.e. occurring despite slowing infusion as discussed above) grade 2 reactions but would not be mandated.

Note that the administration of dexamethasone immediately prior to BMS247550 is unlikely to be effective.

**Note:** An extensive description of the expected adverse events associated with BMS 247550 is provided in Section 5.1 and Appendix A.

##### 6.2 Availability

BMS 247550 is an investigational agent supplied to investigators by the Division of Cancer Treatment and Diagnosis (DCTD), NCI.

BMS 247550 is provided to the NCI under a Clinical Trials Agreement (CTA) between Bristol Myers Squibb and the DCTD, NCI (see Section 10.4).

**6.3 Agent Ordering**

Except in very unusual circumstances, each Participating Institution will order DCTD-supplied investigational agents directly from CTEP. Investigational agents may be ordered by a participating site only after the initial IRB approval for the site has been forwarded by the Coordinating Center to the CTEP PIO.

NCI-supplied agents may be requested by the Protocol Chair (or their authorized designees) at each Participating Institution. Pharmaceutical Management Branch (PMB) policy requires that the agent be shipped directly to the institution where the patient is to be treated. PMB does not permit the transfer of agents between institutions (unless prior approval from PMB is obtained). Completed Clinical Drug Requests (NIH-986) should be submitted to the PMB by fax (301) 480-4612 or mailed to the Pharmaceutical Management Branch, CTEP, DCTD, NCI, 9000 Rockville Pike, EPN Rm. 7149, Bethesda, MD 20892.

**6.4 Agent Accountability**

The Investigator, or a responsible party designated by the investigator, must maintain a careful record of the inventory and disposition of all agents received from DCTD using the NCI Drug Accountability Record Form.

1. **CORRELATIVE/SPECIAL STUDIES**

Genomic DNA and total RNA will be extracted from both mouthwash specimens and PBMCs as a source of normal control cells, which we can utilize to evaluate the occurrence of polymorphisms in tubulin. Where tumor biopsies are available, nucleic acids will also be extracted for the purpose of mutational analysis of tubulin and RT-PCR analysis of additional genes.

1. To evaluate the % microtubule (MT) bundle formation in Peripheral Blood Mononuclear Cells (PBMCs), as a surrogate marker of drug pharmacodynamics.

The mechanism of action of Taxol involves mitotic arrest through microtubule stabilization, which is characterized by the formation of stable bundles or parallel arrays of microtubules and/or the formation of mitotic asters. Since BMS 247550 shares the same mechanism of action as Taxol, we will investigate its effects on the extent of microtubule bundle and mitotic aster formation in pre‑and post‑treatment PBMCs and tumor samples by immunohistochemistry utilizing an alpha‑tubulin antibody. These results will be correlated with pharmacological data at the Phase II dose. This will be important in validating any PK/PD relationships with this class of agent.

2. To determine whether polymorphisms or mutations in tubulin affect response, or toxicity to BMS 247550.

To further define the importance of both β-tubulin isotype expression and frequency of mutations in Class I with clinical response to BMS 247550, we will isolate total RNA from patient tumors (when available) pre‑ (0h) and post‑ treatment (1 and 24h) (Appendix D). Samples will be reverse transcribed into cDNA's using random hexamers. Total protein will also be isolated from samples to correlate RNA and protein expression levels. All total RNA, cDNA and protein lysates will be carefully catalogued and stored at –70ºC. Using sequence‑specific primers that we have designed, β-tubulin isotype cDNA expression levels will be evaluated by real time PCR and transcript levels accurately determined. In addition, the cDNA's from Class I β‑tubulin will be amplified using five nested‑primer sets and the products purified for automatic sequencing. At the Phase II dose of BMS 247550, the response in each patient will be correlated with (a) alterations in β‑tubulin isotype distribution, and (b) the occurrence of polymorphisms / mutations, initially in Class I isotypes. PBMCs and buccal cells (obtained pre-BMS 247550 treatment) will be used to control for naturally occurring polymorphisms in tubulin sequences, or for patients who have acquired tubulin aberrations due to previous chemotherapy.

3. To evaluate whether BMS 247550 efficacy / response is modulated by drug transporters, such as MDR1, MRP, and cMOAT.

Another mechanism for Taxol resistance is the overexpression of P-glyocoprotein. Taxol has a high degree of hydrophobicity and therefore is a substrate for P‑glycoprotein, the product of the MDR1 gene. The multidrug resistance-associated protein, MRP, is a member of the ABC transporter family of genes that regulates cellular drug efflux. Phenotypically, overexpression of MRP confers resistance to anthracyclines, vincristine and etoposide, but not to Taxol. While the preliminary data for BMS 247550 suggests that it retains efficacy in models that overexpress P-glycoprotein, the possibility remains that this compound could induce the overexpression of alternate transporters, therefore contributing to acquired resistance^39,60^. Hence, tumor cells from all evaluable patients will be routinely screened for MDRI and MRP overexpression using RT‑PCR. We will also screen for expression of canalicular multispecific organic-anion transporter (cMOAT), a homologue of MRP whose function is to transport organic anions from hepatocytes into bile^61^. Increased expression of cMOAT has been reported in a variety of human cancer cell lines and in drug resistant cell lines^61^, particularly with cisplatin resistance^62^. It is anticipated that cMOAT will confer resistance to natural product anticancer drugs in a fashion similar to MRP, therefore it seems prudent to assess its expression in tumor cells by RT‑PCR.

4. To evaluate the cytotoxic / growth inhibitory characteristics of BMS 247550.

The mechanism by which Taxol induces cell death is not fully understood, and the role of proteins that regulate apoptosis (e.g. bel‑2 family members, p53, caspases) and modulate Taxol‑induced cytotoxicity requires clarification. The well documented effects of Taxol including microtubule bundle formation and mitotic arrest are not observed at low Taxol concentrations (10nM), although apoptosis is prevalent. It has been suggested that defects in apoptosis-regulating genes are important for the pathogenesis of human cancers^65^, a hypothesis that is supported by the observation that p53 is frequently deleted or mutated in various cancers. In support of this hypothesis, bcl‑2, an inhibitor of apoptosis is overexpressed in a variety of cancer types conferring a survival advantage to cancer cells^66^. Bcl‑2 and bcl‑x proteins prevent apoptosis by blocking cytochrome c release from the mitochondria, by either modulating the ionic imbalances in mitochondria or by binding to the apoptotic protease‑activating factor 1 (Apaf‑1) and preventing downstream caspase activation. Taxol has been shown to bind to bcl-2 inducing its inactivation by hyperphosphorylation and N-terminal cleavage to produce a pro-apoptotic protein that is unable to revert the death stimulus. Hyperphosphorylation of bc1‑2 has also been observed following exposure to other microtubule interacting drugs. We will evaluate the expression of bcl‑2 and bcl-x in pre‑and post‑treatment tumors and PBMC samples using immunofluoresence and Western blotting techniques, taking note of any evidence of hyperphosphorylation in post‑treatment tumor samples. We anticipate that hyperphosphorylation will be difficult to ascertain in vivo since the activation of bcl-2 and bcl‑x is transient, and may be associated with mitotic arrest^67^. Moreover, we will correlate the degree of overexpression of both proteins with clinical outcome. We also plan to quantify the degree of drug-induced cell death in tumor samples pre- and post BMS 247550 administration for evaluable patients, utilizing an antibody that recognizes only the cleaved product of the nuclear repair enzyme PARP (Appendix E).

Other interesting proteins that regulate apoptosis are called inhibitors of apoptosis (IAPs), whose function are to prevent apoptosis by mechanisms involving inhibition of caspases, either directly or indirectly. Survivin is a member if the IAP family that is expressed at the G_2_/M phase of the cell cycle and associates with microtubules. Disruption of the surviving-microtubule interactions results in the loss of survivin's anti‑apoptotic function and increased caspase-3 activity. Hence, survivin is thought to function as a regulator of the G_2_/M checkpoint, and its overexpression in human cancer cells may contribute to the aberrant progression of transformed cells through mitosis^68^. Since the mechanism of activation of BMS 247550 involves mitotic arrest, we will evaluate and compare the expression of survivin in pre‑and post‑treatment tumors using antibodies specific for survivin. In addition, we will assess the interaction of survivin with microtubules in these samples by immunoprecipitating with α- or β-tubulin prior to western blot analysis.

To evaluate the effect of BMS 247550 on cell cycle progression we will monitor the expression of mitotic markers, such as TG3 in tumor biopsies pre- and post-drug treatment.

5. To assess the effect of BMS 247550 on stathmin expression and phosphorylation status.

Stathmin, or p19, is a phosphorylation‑responsive regulator of microtubule dynamics that binds to and sequesters tubulin dimers, resulting in inhibition of microtubule assembly. We have previously shown that the microinjection of recombinant stathmin into COS-7 cells induces loss of microtubules^63^. The phosphorylation status of stathmin fluctuates during the cell cycle, reaching a maximum at mitosis, however the non‑phosphorylated form appears to be responsible, for microtubule destabilization. Our preliminary data indicate that stathmin inhibits the effects of Taxol in an *in vitro* microtubule assembly assay, and also in cells suggesting that endogenous levels of stathmin in human tumors may determine sensitivity to Taxol and possibly other microtubule interacting agents. Recent evidence suggests that Raf‑1 kinase associates with stathmin, and we have demonstrated that there is a dose and time‑dependent phosphorylation of stathmin following Taxol exposure^64^. The depletion of Raf‑1 kinase is associated with decreased Taxol‑induced stathmin phosphorylation levels, a phenomenon that may be partially mediated by MAP kinases. It is reasonable to assume that BMS 247550 will exert similar effects on stathmin and we anticipate that there may be a strong correlation between stathmin expression status and BMS 247550 efficacy. We will determine the expression status of stathmin in pre- and post-*­*treatment patient tumors using RT‑PCR to ascertain mRNA expression levels, and at the protein level, using specific antibodies which recognize either phosphorylated or non-phosphorylated epitopes and analyzing samples utilizing non‑denaturing electrophoresis (Appendix E). This information will be used to ascertain whether endogenous stathmin expression correlates with clinical response to BMS 247550, or whether the distribution of phosphorylated and non‑phosphorylated stathmin is modulated following treatment. The storage of all tumor extracts at ‑70C will facilitate future expression studies of other microtubule‑associated proteins such as MAP4 and E-MAP-115, both of which regulate microtubule dynamics.

6. To correlate progression through G2 and mitosis with response to BMS 247550:

Phosphorylation of histone (H3) correlates with chromatin condensation at mitosis. A new monoclonal antibody (anti-H3-P) was developed that recognizes phosphorylated H3 (H3-P). Mitotic cells, from prophase to telephase, reacted with anti-H3-P. The analysis of phosphorylation of H3 before and afterBMS-247550, when combined with multi-parameter analysis of cycle position and expression of other proteins offer new possibilities to study molecular mechanisms associated with the G2 to M transition and chromatin condensation. In addition, we will examine pre- and post-treatment available tissues for mitotic phosphorylation recognized by the antibody MPM2.

**NYU 00-57 STUDY CALENDAR**

Baseline evaluations are to be conducted no more than 1 week prior to administration of study agent. Scans and x-rays must be done no more than 4 weeks prior to the start of therapy. If the patient’s condition is deteriorating, laboratory evaluations should be repeated no more than 48 hours prior to initiation of the next cycle of therapy.

|  | **Pre-Study** | **Each cycle** | | **Every 2 cycles** | **Off Study** |
| --- | --- | --- | --- | --- | --- |
|  |  | **D1, D8, D15** | **Prior to next cycle (D22-27)** |  |  |
| BMS 247550 |  | X |  |  |  |
| **Informed consent** | X |  |  |  |  |
| **Demographics** | X |  |  |  |  |
| **Medical history** | X |  | X |  |  |
| **Physical exam** | X |  | X |  |  |
| **Vital signs** | X | X |  |  |  |
| **Height / Weight** | X | X |  |  |  |
| **Medications** | X |  | X |  |  |
| **Performance status** | X |  | X |  | X |
| **Hb, ANC, plts*** | X | X | X |  |  |
| **Serum chemistry**** | X |  | X |  |  |
| **EKG (as indicated)** | X |  |  |  |  |
| **B-HCG** | X |  |  |  |  |
| **Adverse Events** |  | X | X |  | X |
| **Tumor Measurements** |  |  |  | X |  |
| **Collection of PBPC’s (1 & 24h: cycles 1 &2)** | X | X |  |  |  |
| **Tumor Biopsy (1 & 24h cycles 1 &2)** | X | X |  |  | X |

* If a patient develops Grade 4 neutropenia, CBC’s will be checked 3 times per week until resolution.

** Electrolytes, calcium, magnesium, liver function tests and LDH.

**9. MEASUREMENT OF EFFECT**

For the purposes of this study, patients should be re-evaluated for response every 2 cycles. In addition to a baseline scan, confirmatory scans should also be obtained *not less than 4* weeks following initial documentation of objective response.

**9.1 Definitions**

Response and progression will be evaluated in this study using the new international criteria proposed by the Response Evaluation Criteria in Solid Tumors (RECIST) Committee [*JNCI* 92(3):205-216, 2000]. Changes in only the largest diameter (unidimensional measurement) of the tumor lesions are used in the RECIST criteria. Note: Lesions are either measurable or non-measurable using the criteria provided below. The term “evaluable” in reference to measurability will not be used because it does not provide additional meaning or accuracy.

**9.1.1 Measurable disease**

Measurable lesions are defined as those that can be accurately measured in at least one dimension (longest diameter to be recorded) as ≥20 mm with conventional techniques (PET, CT, MRI, x-ray) or as ≥10 mm with spiral CT scan. All tumor measurements must be recorded in millimeters.

**Note:** Tumor lesions that are situated in a previously irradiated area will be considered measurable only if there has been documented progression of the lesion since completion of radiotherapy.

Clinical lesions

Clinical lesions will only be considered measurable when they are superficial (e.g., skin nodules and palpable lymph nodes). In the case of skin lesions, documentation by color photography, including a ruler to estimate the size of the lesion, is recommended.

Chest x-ray

Lesions on chest x-ray are acceptable as measurable lesions when they are clearly defined and surrounded by aerated lung. However, CT is preferable.

Conventional CT and MRI

These techniques should be performed with cuts of 10 mm or less in slice thickness contiguously. Spiral CT should be performed using a 5 mm contiguous reconstruction algorithm. This applies to tumors of the chest, abdomen, and pelvis. Head and neck tumors and those of extremities usually require specific protocols.

Ultrasound (US)

When the primary endpoint of the study is objective response evaluation, US should not be used to measure tumor lesions. It is, however, a possible alternative to clinical measurements of superficial palpable lymph nodes, subcutaneous lesions, and thyroid nodules. US might also be useful to confirm the complete disappearance of superficial lesions usually assessed by clinical examination.

Endoscopy, Laparoscopy

The utilization of these techniques for objective tumor evaluation has not yet been fully and widely validated. Their uses in this specific context require sophisticated equipment and a high level of expertise that may only be available in some centers. Therefore, the utilization of such techniques for objective tumor response should be restricted to validation purposes in reference centers. However, such techniques may be useful to confirm complete pathological response when biopsies are obtained.

Tumor markers

Tumor markers alone cannot be used to assess response. If markers are initially above the upper normal limit, they must normalize for a patient to be considered in complete clinical response. Specific additional criteria for standardized usage of prostate-specific antigen (PSA) and CA-125 response in support of clinical trials are being developed.

Cytology, Histology

These techniques can be used to differentiate between partial responses (PR) and complete responses (CR) in rare cases (e.g., residual lesions in tumor types, such as germ cell tumors, where known residual benign tumors can remain).

The cytological confirmation of the neoplastic origin of any effusion that appears or worsens during treatment when the measurable tumor has met criteria for response or stable disease is mandatory to differentiate between response or stable disease (an effusion may be a side effect of the treatment) and progressive disease.

**9.1.2 Non-measurable disease**

All other lesions (or sites of disease), including small lesions (longest diameter <20 mm with conventional techniques or <10 mm using spiral CT scan), are considered non-measurable disease. Bone lesions, leptomeningeal disease, ascites, pleural/pericardial effusions, lymphangitis cutis/pulmonis, inflammatory breast disease, abdominal masses (not followed by CT or MRI), and cystic lesions are all non-measurable.

**9.1.3 Target lesions**

All measurable lesions up to a maximum of five lesions per organ and 10 lesions in total representative of all involved organs should be identified as **target lesions** and recorded and measured at baseline. Target lesions should be selected on the basis of their size (lesions with the longest diameter) and their suitability for accurate repeated measurements (either by imaging techniques or clinically). A sum of the longest diameter (LD) for all target lesions will be calculated and reported as the baseline sum LD. The baseline sum LD will be used as reference by which to characterize the objective tumor response.

**9.1.4 Non-target lesions**

All other lesions (or sites of disease) should be identified as **non-target lesions** and should also be recorded at baseline. Non-target lesions include measurable lesions that exceed the maximum numbers per organ or total of all involved organs as well as non-measurable lesions. Measurements of these lesions are not required, but the presence or absence of each should be noted throughout follow-up.

**9.2 Guidelines for Evaluation of Measurable Disease**

All measurements should be taken and recorded in metric notation using a ruler or calipers. All baseline evaluations should be performed as closely as possible to the beginning of treatment and never more than 4 weeks before the beginning of the treatment.

The same method of assessment and the same technique should be used to characterize each identified and reported lesion at baseline and during follow-up. Imaging-based evaluation is preferred to evaluation by clinical examination when both methods have been used to assess the antitumor effect of a treatment.

**9.3** **Response Criteria**

**9.3.1 Evaluation of target lesions**

Complete Response (CR)

Disappearance of all target lesions

Partial Response (PR)

At least a 30% decrease in the sum of the longest diameter (LD) of target lesions, taking as reference the baseline sum LD

Progressive Disease (PD)

At least a 20% increase in the sum of the LD of target lesions, taking as reference the smallest sum LD recorded since the treatment started or the appearance of one or more new lesions

Stable Disease (SD)

Neither sufficient shrinkage to qualify for PR nor sufficient increase to qualify for PD, taking as reference the smallest sum LD since the treatment started

**9.3.2 Evaluation of non-target lesions**

Complete Response (CR)

Disappearance of all non-target lesions and normalization of tumor marker level

Note: If tumor markers are initially above the upper normal limit, they must normalize for a patient to be considered in complete clinical response.

Incomplete Response/Stable Disease (SD)

Persistence of one or more non-target lesion(s) and/or maintenance of tumor marker level above the normal limits

Progressive Disease (PD)

Appearance of one or more new lesions and/or unequivocal progression of existing non-target lesions

Although a clear progression of “non-target” lesions only is exceptional, in such circumstances the opinion of the treating physician should prevail, and the progression status should be confirmed at a later time by the review panel (or study chair).

**9.3.3 Evaluation of best overall response**

The best overall response is the best response recorded from the start of the treatment until disease progression/recurrence (taking as reference for progressive disease the smallest measurements recorded since the treatment started). The patient's best response assignment will depend on the achievement of both measurement and confirmation criteria (see section 9.3.1).

| Target Lesions | Non-Target Lesions | New Lesions | Overall Response |
| --- | --- | --- | --- |
| CR | CR | No | CR |
| CR | Incomplete response/SD | No | PR |
| PR | Non-PD | No | PR |
| SD | Non-PD | No | SD |
| PD | Any | Yes or No | PD |
| Any | PD | Yes or No | PD |
| Any | Any | Yes | PD |

**Note:**

- Patients with a global deterioration of health status requiring discontinuation of treatment without objective evidence of disease progression at that time should be classified as having “symptomatic deterioration.” Every effort should be made to document the objective progression, even after discontinuation of treatment.
- In some circumstances, it may be difficult to distinguish residual disease from normal tissue. When the evaluation of complete response depends on this determination, it is recommended that the residual lesion be investigated (fine needle aspirate/biopsy) before confirming the complete response status.

**9.4 Confirmatory Measurement / Duration of Response**

**9.4.1 Confirmation**

To be assigned a status of PR or CR, changes in tumor measurements must be confirmed by repeat assessments that should be performed no less than 4 weeks after the criteria for response are first met. In the case of SD, follow-up measurements must have met the SD criteria at least once after study entry at a minimum interval of 6 weeks (see section 9.3.3).

**9.4.2 Duration of overall response**

The duration of overall response is measured from the time measurement criteria are met for CR or PR (whichever is first recorded) until the first date that recurrent or progressive disease is objectively documented (taking as reference for progressive disease the smallest measurements recorded since the treatment started).

The duration of overall CR is measured from the time measurement criteria are first met for CR until the first date that recurrent disease is objectively documented.

**9.4.3 Duration of stable disease**

Stable disease is measured from the start of the treatment until the criteria for progression are met, taking as reference the smallest measurements recorded since the treatment started.

**9.5 Time to Progression**

Time to progression is defined as the time from the first day of treatment with BMS 247550 until the first documentation of disease progression.

**9.6**  **Response Review**

All responses will be reviewed by an expert(s) independent of the study at the study’s completion. Simultaneous review of the patients’ files and radiological images will be performed.

**10. REGULATORY AND REPORTING REQUIREMENTS**

- 1. **Patient Registration**

Prior to registration, each Participating Institution must forward a copy of its IRB approval and approved consent form to the Coordinating Center and to the NCI Protocol Information Office (see Multicenter Guidelines, section 10.4).

To register a patient, a completed eligibility checklist should be sent to the study data manager at NYU (see Appendix F). Completed eligibility checklists may be forwarded electronically to **both** the Protocol Chair (anna.pavlick@med.nyu.edu) and to the Study Coordinator (dellec02@med.nyu.edu), or faxed to (212) 263-8210.

Following central eligibility verification, registration will be confirmed by allocation of a patient identification number.

**10.2 Adverse Event Reporting**

- - 1. **Common Toxicity Criteria**

Adverse events (AE) will use the descriptions and grading scales found in the revised NCI Common Toxicity Criteria (CTC). This study will utilize the CTC version 2.0 for adverse event reporting. All appropriate treatment areas should have access to a copy of the CTC version 2.0, which is available at <http://ctep.info.nih.gov/CTC3/>.

- - 1. **Expedited Reporting Guidelines**

**EXPEDITED ADVERSE EVENT REPORTING**

**PHASE II AND III STUDIES WITH INVESTIGATIONAL AGENTS**

**SUPPLIED UNDER CTEP-SPONSORED IND’S**

Expedited Reporting for Phase II Studies (Including hospitalization defined in bullet 1 below)

| **Unexpected Event** | | **Expected Event** | |
| --- | --- | --- | --- |
| **Grades 2 – 3**  **Attribution of Possible, Probable or Definite** | **Grades 4 and 5**  **Regardless of Attribution** | **Grades 1 - 3** | **Grades 4 and 5**  **Regardless of Attribution** |
| Expedited report within 10 working days.  (Grade 1 Adverse Event Expedited Reporting NOT required.) | Report by phone to IDB within 24 hrs. Expedited report to follow within 10 working days.  This includes all deaths witnin 30 days of the last dose of treatment with an investigational agent regardless of attribution.  Any late death attributed to the agent (possible, probable, or definite) should be reported within 10 working days. | Adverse Event Expedited Reporting NOT required. | Expedited report, including Grade 5 Aplasia in leukemia patients, within 10 working days.  This includes all deaths within 30 days of the last dose of treatment with an investigational agent regardless of attribution.  Any late death attributed to the agent (possible, probable, or definite) should be reported within 10 working days.  Grade 4 Myelosuppression or other Grade 4 events that do not require expedited reporting will be specified in the protocol. |

A table showing the expected adverse events associated with BMS 247550 can be found in section 5.1 and in Appendix A.

- For **Hospitalization** only- Any medical event equivalent to CTC Grade 3,4,5 which precipitated hospitalization 9or prolongation of existing hospitalization ) must be reported regardless of expected or unexpected and attribution.
- Telephone reports to the Investigational Drug Branch at 1 (301) 230 2330 available 24 hours daily (recorder between 5pm and 9am EST).
- Expedited reports are to be submitted using AdEERS or the paper templates available at [http://ctep.info.nih.gov](http://ctep.info.nih.gov/). The NCI guidelines for expedited adverse event reporting are also available at this site.
- A list of agent specific expected adverse events (ASAEL) can be found in this protocol.

**10.2.3 Forms**

AE’s should be reported electronically using the AdEERS system, full details of which are available at <http://ctep.info.nih.gov/AdEERS/>.

If electronic reporting is not possible within the required timeframe, reports should be made on the DCTD Form for Reporting AEs Occurring with Investigational Agents Obtained from the NCI. This form can be downloaded from the CTEP home page <http://ctep.info.nih.gov/InfoForms/default.htm>. Cases of secondary AML/MDS are to be reported using the NCI/CTEP Secondary AML/MDS Report Form.

**10.3 Data Reporting**

Data will be reported electronically to the Coordinating Center, using an Excel spreadsheet template that will be provided to the Participating Institutions.

Data updates should be forwarded to the Coordinating Center within 2 weeks of completion of each treatment cycle.

This study will be monitored by the Clinical Data Update System (CDUS) version 2.0. Cumulative CDUS data will be submitted electronically to CTEP by the Coordinating Center on a quarterly basis. Reports are due January 31, April 30, July 31, and October 31. Instructions for submitting data using the CDUS can be found on the CTEP home page (<http://ctep.info.nih.gov/CtepInformatics/CDUS/Default.htm>). Sample data collection forms are contained in Appendix G.

**10.4 CTEP Multicenter Guidelines**

**10.4.1 Responsibilities of the Protocol Chair**

- The Protocol Chair will be the single liaison with the CTEP Protocol and Information Office (PIO). The Protocol Chair is responsible for the coordination, development, submission, and approval of the protocol as well as its subsequent amendments. The protocol must not be rewritten or modified by anyone other than the Protocol Chair. There will be only one version of the protocol, and each Participating Institution will use that document. The Protocol Chair is responsible for assuring that all Participating Institutions are using the correct version of the protocol.
- The Protocol Chair is responsible for the overall conduct of the study at all Participating Institutions and for monitoring its progress. All reporting requirements to CTEP are the responsibility of the Protocol Chair.
- The Protocol Chair is responsible for the timely review of Adverse Events (AE) to assure safety of the patients.
- The Protocol Chair will be responsible for the review of and timely submission of data for study analysis.

**10.4.2 Responsibilities of the Coordinating Center**

- Each Participating Institution will have an appropriate assurance on file with the Office for Protection from Research Risks (OPRR), NIH. The Coordinating Center is responsible for assuring that each Participating Institution has an OPRR assurance and must maintain copies of IRB approvals from each participating site.
- Prior to the activation of the protocol at each Participating Institution, an OPRR form 310 (documentation of IRB approval) must be submitted to the CTEP PIO.
- The Coordinating Center is responsible for central patient registration. The Coordinating Center is responsible for assuring that IRB approval has been obtained at each participating site prior to the first patient registration from that site.
- The Coordinating Center is responsible for the preparation of all submitted data for review by the Protocol Chair.
- The Coordinating Center will maintain documentation of AE reports. There are two options for AE reporting: (1) Participating Institutions may report directly to CTEP with a copy to the Coordinating Center, or (2) Participating Institutions report to the Coordinating Center who in turn report to CTEP. The Coordinating Center will submit AE reports to the Protocol Chair for timely review.
- Audits may be accomplished in two ways: (1) source documents and research records for selected patients are brought from participating sites to the Coordinating Center for NCI audit , and (2) selected patient records will be audited on-site at participating sites yearly. The NCI will be perform all audits at the Coordinating Center, subsequently the Coordinating Center will be responsible for having all source documents, research records, all IRB approval documents, NCI Drug Accountability Record forms, patient registration lists, response assessments scans, x-rays, etc. available for the audit.

**10.5 Clinical Trials Agreement (CTA)**

The agent, BMS 247550, used in this protocol is provided to the NCI under a Clinical Trials Agreement (CTA) between Bristol Myers Squibb [hereinafter referred to as “Collaborator”] and the NCI Division of Cancer Treatment and Diagnosis. Therefore, the following obligations/guidelines, in addition to the provisions in the “Intellectual Property Option to Collaborator” terms of award modifications, apply to the use of BMS 247550 in this study:

1. BMS 247550 may not be used for any purpose outside the scope of this protocol, nor can BMS 247550 be transferred or licensed to any party not participating in the clinical study. Collaborator data for BMS 247550 are confidential and proprietary to Collaborator and shall be maintained as such by the investigators.

2. For a clinical protocol where there is an investigational agent used in combination with (an)other investigational agent(s), each the subject of different CTAs, the access to and use of data by each Collaborator shall be as follows (data pertaining to such combination use shall hereinafter be referred to as "Multi-Party Data”.):

a. NCI must provide all Collaborators with prior written notice regarding the existence and nature of any agreements governing their collaboration with NIH, the design of the proposed combination protocol, and the existence of any obligations which would tend to restrict NCI's participation in the proposed combination protocol.

b. Each Collaborator shall agree to permit use of the Multi-Party Data from the clinical trial by any other Collaborator solely to the extent necessary to allow said other Collaborator to develop, obtain regulatory approval, or commercialize its own investigational agent.

c. Any Collaborator having the right to use the Multi-Party Data from these trials must agree in writing prior to the commencement of the trials that it will use the Multi-Party Data solely for development, regulatory approval, and commercialization of its own investigational agent.

3. Clinical Trial Data and Results and Raw Data developed under a CTA will be made available exclusively to Collaborator, the NCI, and the FDA, as appropriate.

4. When a Collaborator wishes to initiate a data request, the request should first be sent to the NCI, who will then notify the appropriate investigators (Group Chair for Cooperative Group studies, or PI for other studies) of Collaborator's wish to contact them.

5. Any data provided to Collaborator for Phase 3 studies must be in accordance with the guidelines and policies of the responsible Data Monitoring Committee (DMC), if there is a DMC for this clinical trial.

1. Any manuscripts reporting the results of this clinical trial should be provided to CTEP for immediate delivery to Collaborator for advisory review and comment prior to submission for publication. Collaborator will have 30 days from the date of receipt for review. Collaborator shall have the right to request that publication be delayed for up to an additional 30 days in order to ensure that Collaborator’s confidential and proprietary data, in addition to Collaborator’s intellectual property rights, are protected. Copies of abstracts should be provided to CTEP for forwarding to Collaborator for courtesy review as soon as possible and preferably at least three (3) days prior to submission, but in any case, prior to presentation at the meeting or publication in the proceedings. Copies of any manuscript and/or abstract should be sent to:

Regulatory Affairs Branch, CTEP, DCTD, NCI

Executive Plaza North, Room 718

Bethesda, Maryland 20892

FAX 301-402-1584

The Regulatory Affairs Branch will then distribute them to Collaborator. No publication, manuscript or other form of public disclosure shall contain any of Collaborator’s confidential/ proprietary information.

**11. STATISTICAL CONSIDERATIONS**

**11.1 Endpoints**

Primary endpoint:

- Response rate (RR)

Secondary endpoints:

- Time to progression (TTP)
- Toxicity
- Correlative Data

**11.2 Study Design / Stratification Factors**

The three-stage optimal design for phase II clinical trials proposed by Ensign et al.^69^ will be used in each subgroup. A beta error probability of 0.10 (lower than the commonly used 0.20) was chosen because of the paucity of new drugs that may be active in melanoma.

**Subgroup A: Chemotherapy naïve:**

A response rate of 25% or greater would justify further study of this treatment regimen whereas a rate of 10% or less would be considered uninteresting.

Patient accrual will proceed in 3 stages as follows:

| Accrual stage | Number of patients to be accrued in stage | Cumulative number of patients (n) accrued at end of stage | Reject drug if response rate ≤ r^1^/n |
| --- | --- | --- | --- |
| 1  2  3 | 11  18  21 | 11  29  50 | 0/11  3/29  7/50 |

^1^ r = cumulative number of responses observed

For this design both the alpha and beta error probabilities are 0.10. The overall probability that the trial will be stopped early when the true response rate is 10% is 0.70.

**Subgroup B: DTIC/Temozolomide pretreated:**

A response rate of 20% or greater would justify further study of this treatment regimen whereas a rate of 5% or less would be considered uninteresting.

Patient accrual will proceed in 3 stages as follows:

| Accrual stage | Number of patients to be accrued in stage | Cumulative number of patients (n) accrued at end of stage | Reject drug if response rate ≤ r^1^/n |
| --- | --- | --- | --- |
| 1  2  3 | 12  13  13 | 12  25  38 | 0/12  1/25  3/38 |

^1^ r = cumulative number of responses observed

For this design both the alpha and beta error probabilities are 0.10. The overall probability that the trial will be stopped early when the true response rate is 5% is 0.72.

**11.3 Sample Size/Accrual Rate**

A total sample size of 23-88 evaluable patients is planned. Accrual of 4-5 patients per month is expected, so that the study should be completed in at most 24 months.

**11.4 Analysis of Secondary Endpoints**

Median time to progression will be described for each subgroup.

Related toxicities will be described.

**11.5 Reporting and Exclusions**

**11.5.1 Toxicity**

All patients will be evaluable for toxicity from the time of their first treatment with BMS 247550.

- - 1. **Evaluation of response**

All patients included in the study must be assessed for response to treatment, even if there are major protocol treatment deviations or if they are ineligible. Each patient will be assigned one of the following categories: 1) complete response, 2) partial response, 3) stable disease, 4) progressive disease, 5) early death from malignant disease, 6) early death from toxicity, 7) early death because of other cause, or 9) unknown (not assessable, insufficient data). [Note: By arbitrary convention, category 9 usually designates the “unknown” status of any type of data in a clinical database.]

All of the patients who met the eligibility criteria should be included in the main analysis of the response rate. Patients in response categories 4-9 should be considered as failing to respond to treatment (disease progression). Thus, an incorrect treatment schedule or drug administration does not result in exclusion from the analysis of the response rate. Precise definitions for categories 4-9 will be protocol specific.

All conclusions should be based on all eligible patients. Subanalyses may then be performed on the basis of a subset of patients, excluding those for whom major protocol deviations have been identified (e.g., early death due to other reasons, early discontinuation of treatment, major protocol violations, etc.). However, these subanalyses may not serve as the basis for drawing conclusions concerning treatment efficacy, and the reasons for excluding patients from the analysis should be clearly reported. The 95% confidence intervals should also be provided.

**12. REFERENCES**

1. Roth A and Kirkwood J: Melanoma: Classic approaches to management and current integration of biologic response modifiers. Current Concepts Oncol. 9:1-11. 1988.

2. Houghton AN, Legha S, Bajorin DF: Chemotherapy for metastatic melanoma, in *Cutaneous Melanoma*, Balch C, Houghton A, Milton, Sober, Soong (eds). 2^nd^ Edition, J.B. Lippincott, Philadelphia, pp. 498-508, 1992.

3. Middleton MR, Gore M, Tilgen W, et al.: Randomized, phase III study of temozolomide (TMZ) versus dacarbazine (DTIC) in the treatment of patients with advanced, metastatic melanoma. J Clin Oncol. 18:158-166, 2000. See also Cohen MH and Johnson JR: Temozolomide for advanced metastatic melanoma (letter), J Clin Oncol. 18:2185, 2000.

4. Legha SS, Ring S, Papadopoulos N, Plager C. Chawla S, Benjamin R: A prospective evaluation of a triple‑drug regimen containing cisplatin, vinblastine and DTIC (CVD) for metastatic melanoma. Cancer 64:2024, 1989.

5. DelPrete SA, Maurer LH, O'Donnell J: Combination chemotherapy with cisplatin, carmustine, dacarbazine and tamoxifen in metastatic melanoma. Cancer Treat Rep. 69:1403. 1984.

6. Buzaid AC, Legha S, Winn R, Belt R, et al.: Cisplatin (C),vinblastine (V), and dacarbazine (D) (CVD) versus dacarbazine alone in metastatic melanoma: Preliminary results of a phase III cancer community oncology program (CCOP) trial. Proc Am Soc Clin Oncol. 12:389, 1993.

7. Cocconi G, Bella M, Calabresi F, et al.: Treatment of metastatic malignant melanoma with dacarbazine plus tamoxifen. N Engl J Med. 327:516-523, 1992.

8. Falkson, CI, Ibrahim, J, Kirkwood J, Blum, R: A randomized phase III trial of dacarbazine (DTIC) versus DTIC + interferon alpha-2b (IFN) versus DTIC + tamoxifen (TMX) versus DTIC + IFN + TMX in metastatic malignant melanoma: an ECOG trial. Proc Am Soc Clin Oncol. 15:435, 1996.

9. McClay E, Mastrangelo M, Bellet R, et al.: An effective chemo/hormonal therapy regimen for the treatment of disseminated malignant melanoma. Proc Am Soc Clin Oncol. 8:282, 1989.

10. Kirkwood JM: Studies of interferon in the therapy of melanoma. Semin Oncol. (suppl 7) 18:83-89, 1991.

11. Sparano JA and Dutcher JP: Interleukin-2 for the treatment of advanced melanoma. Hematol Oncol. Annals 1:279-285, 1993.

12. Dutcher JP, Creekmore S, Weiss GR, et al.: Phase II study of high-dose interleukin-2 and lymphokine activated killer cells in patients with metastatic malignant melanoma. J Clin Oncol. 7:477-485, 1989.

13. Parkinson DR, Abrams JS, Wiernik PH, et al.: Interleukin-2 therapy in patients with metastatic malignant melanoma: a phase II study. J Clin Oncol. 8:1650‑1656, 1990.

14. Rosenberg SA, Yang JC, Topalian SL: Treatment of 283 consecutive patients with metastatic melanoma or renal cell cancer using high-dose bolus interleukin-2. JAMA 907-913, 1994.

15. Rowinsky EK, Cazenave LA, Donehower RC. Paclitaxel: A novel investigational antimicrotubule agent. J Natl Cancer Inst 1990; 82(15):1247-1259.

16. Schiff PB, Fant J, Horwitz SB. Promotion of microtubule assembly in vitro by taxol. Nature 1979: 277;665-7

17. Schiff PB, Horwitz SB. Taxol stabilizes microtubules in mouse fibroblast cells. Proc. Natl. Acad. Sci. USA 1980: 77;1561-5

18. Schiff PB, Horwitz SB. Taxol assembles tubulin in the absence of exogenous guanosine 5’triphosphate or microtubule-associated proteins. Biochemistry 1981: 20;3247-52

1. Rowinsky EK, Donehower RC. Taxol. New Engl J Med 1995: 332;1004-1014

20. Rowinsky EK. The development and clinical utility of the taxane class of antimicrotubule chemotherapy agents. Ann. Rev. Med. 1997: 48;353-74

1. Luduena RF. Multiple forms of tubulin: different gene products and covalent modifications. Int. Rev Cytol 1998: 178;207-275
2. Kirshner MW. Microtubule assembly and nucleation. In Rev Cytol 1978: 54;1-71
3. Wilde CD, Crowther CE, Cripe TP et al. Evidence that a human beta-tubulin pseudogene is derived from its corresponding mRNA. Nature 1982: 297;83-84
4. Lopata MA, Cleveland DW. In vivo microtubules are copolymers of available betatubulin isotypes: localization of each of six vertebrate beta-tubulin isotypes using polyclonal antibodies elicited by synthetic peptide antigens. J Cell Biol. 1994: 105;1707-1720
5. Lewis SA, Gu W, Cowan NJ. Free intermingling of mammalian beta-tubulin isotypes among functionally distinct microtubules. Cell 1987: 49; 539-548
6. Sullivan KF, Cleveland DW. Identification of conserved isotype defining variable region sequences for four vertebrate beta-tubulin polypeptide classes. Proc. Natl. Acad. Sci USA 1986: 83;4327-4331
7. Nogales E, Wolf SG, Downing KH. Structure of the alpha beta tubulin dimer by electron crystallography. Nature 1998: 391;199-203
8. Dumontet C, Sikic BI. Mechanism of action of and resistance to antitubulin agents: microtubule dynamics, drug transport, and cell death. J Clin Oncol. 1999: 17;1061-1070
9. Fan W. Possible mechanisms of paclitaxel-induced apoptosis, Biochem. Pharmacol. 1999: 57;1215-21.
10. Rao S, Krauss NE, Heerding JM, Swindell CS, Ringel 1, Orr GA, Horwitz SB. 3’-(p-azidobenzamido) Taxol photolabels the N-terminal 31 amino acids of beta-tubulin. J. Biol. Chem. 1994: 269;3132-4
11. Rao S, Orr GA, Chaudhary AG, Kingston DG, Horwitz SB. Characterization of the taxol binding site on the microtubule. 2-(m-Azidobenzoyl)Taxol photolabels a peptide (amino acids 217-231) of beta-tubulin. J. Biol. Chem. 1995: 270(35);202-8
12. Rodl DJ, Janes RW, Sanganee HJ, Holton RA, Wallace BA, Makowski L. Screening of a library of phage-displayed peptides identifies human bcl-2 as a taxol-binding protein. J. Mol. Biol. 1999: 285(l);197-203
13. Bhat N, Perera PY, Carboni JK Blanco J, Golenbock DT, Mayadas TN, Vogel SN. Use of a photoactivatable taxol analogue to identify unique cellular targets in murine macrophages: identification of murine CD18 as a major taxol-binding protein and a role for Mac-I in taxol-induced gene expression. J Immunol. 1999: 162;7335-42
14. Cabral F, Barlow SB. Mechanisms by which mammalian cells acquire resistance to drugs that affect microtubule assembly. FASEB J 1989: 3;1593-1599
15. Minotti A-K Barlow SB, Cabral F. Resistance to antimitotic drugs in Chinese hamster ovary cells correlates with changes in the level of polymerized tubulin. J. Biol. Chem. 1991: 266;3987-3994
16. Kavallaris M, Burkhart CA, Horwitz SB. Antisense oligonucleotides to class III beta-tubulin sensitize drug-resistant cells to taxol. Br J Cancer: Jun 1999: 80; 1020-5
17. Haber M, Burkhart CA, Regi DL, Madafiglio J, Norris MD, Horwitz SB. Altered expression of M beta 2, the class II beta-tubulin isotype in a murine J774.2 cell line with a high level of taxol resistance. J. Biol. Chem: 1995: 270;1269-75
18. Weisen K and Horwitz SB. 1999; (unpublished observation)
19. Kavallaris M, Kuo DYS, Burkhart CA, Regl DL, Norris MD, Haber M, Horwitz SB. Taxol resistant epithelial ovarian tumors are associated with altered expression of specific beta-tubulin isotypes. J. Clin. Invest: 1997: 100;1282-93.
20. Dumontet C, Jaffrezou JP, Tsuchiya E et al. Resistance to microtubule-targeted cytotoxins in a K562 leukemia cell variant associated with altered tubulin expression and polymerization. Elec. J. Oncol. 1998: 2;44-54.
21. Giannakakou P, Sackett DL, Kang YK, Zhan Z, Buters JT, Fojo T, Poruchynsky MS. Paclitaxel-resistant human ovarian cancer cells have mutant beta-tubulins that exhibit impaired paclitaxel-driven polymerization. J. Biol. Chem. 1997: 272;17118-25
22. Gonzalez-Garay ML, Chang L, Blade K, Menick DR, Cabral F. A beta-tubulin leucine cluster involved in microtubule assembly and paclitaxel resistance. J. Biol. Chem. 1999: 274;23875-82
23. Giannakakou P, Gussio R, Nogales E, Downing KH, Zaharevitz D, Bollbuck B, Poy G, Sackett D, Nicolaou KC, Fojo T. A common pharmacophore for epothilone and taxanes: Molecular basis for drug resistance conferred by tubulin mutations in human cancer cells. Proc. Nat. Acad. Sci. USA 1999: 10;1073-9
24. Monzo M, Rosell R, Sanchez J-J, Lee J-S, aBrate A, Gonzalez-Larriba JS, Alberola V, Lorenzo JC, Nunez L, Ro JY and Martin C. Paclitaxel resistance in non-small cell lung cancer associated with beta-tubulin gene mutations. J. Clin Oncol 1999: 17; 1786-1793.
25. Hofie G, Bedord N, Steinmetz H, Schomburg D, Gerth K, Reichenbach H. Epothilone A and B - novel 16-membered macrolides and cytotoxic activity: isolation, crystal structure, and conformation in solution. Angew. Chem. Int. Ed Engl. 1999: 25;1567-1569.
26. Bollag DM, McQueney PA, Zhu J, Hensens O, Koupal L, Liesch J, Goetz M, Lazarides E, Woods CM. Epothilones, a new class of microtubule-stabilizing agents with a taxol-like mechanism of action. Cancer Res. 1995; 55:2325-33.
27. Kowalski RJ, Giannakakou P, Hamel E. Activities of the microtubule-stabilizing agents Epothilones A and B with purified tubulin and in cells resistant to paclitaxel (Taxol ®). J. Biol. Chem. 1997: 272;2534-41
28. Muhlradt PF, Sasse F. Epothilone B stabilizes microtubulin of macrophages like taxol without showing taxol-like endotoxin activity. Cancer Res. 1997: 57;3344-6

49. BMS 247550. Bristol-Myers Squibb Investigator Brochure, Version #2, 2000.

50. Summary of preclinical pharmacokinetics of BMS 247550. Bristol-Myers Squibb Pharmaceutical Research Institute, 1999; BMS Document Control Number 910074594.

51. Toxicokinetic report for BMS 247550: Single dose intravenous toxicity study in rats (study 99612). Bristol-Myers Squibb Pharmaceutical Research Institute, 1999; BMS Document Control No. 920000516.

52. Toxicokinetic report for BMS 247550: Single dose intravenous toxicity study in dogs (study 99617). Bristol-Myers Squibb Pharmaceutical Research Institute, 1999; BMS Document Control No. 920000574.

1. Su D-S, Balog A, Meng D, Bertinato P, Danishefsky S, Zheng Y-H, Chou T-C, He L and Horwitz SB. Structure-activity relationships of the epothilones and the first in vivo comparison with taxol. Agnew. Chem. Int. Ed. Engl. 1997: 36;2093-2096
2. Stroun M, Maurice P, Vasioukhin V, Lyautey J, Lederrey C, Lefort F, Rossier A, Chen XQ, Anker P. The origin and mechanism of circulating DNA. Ann NY Acad Sci 2000: 906;161-8
3. Anker P, Mulcahy H, Chen XQ, Stroun M. Detecton of circulating tumour DNA in the blood (plasma/serum) of cancer patents. Cancer Metastasis Rev 1999: 18(l);65-73
4. Taxol® Product information in. In: Physician’s Desk Reference Edition 53. Montvale NJ, Medical Economics Company, Inc., 1999, p. 798-803.
5. Chen X, Bonnefoi H, Diebold-Berger S, Lyautey J, Lederrey C, Faltin-Traub E, Stroun M, Anker P. Detecting tumor-related alterations in plasma or serum DNA of patents diagnosed with breast cancer. Clin Cancer Res 1999: 5(9);2297-303
6. Mulcahy HE, Lyautey J, Lederrey C, Chen XQ, Lefort F, Vasioukhin V, Anker P, Alstead EM, Farthing MJ, Stroun M. Plasma DNA K-ras mutations in patents with gastrointestinal malignancies. Ann NY Acad Sci 2000: 906;25-8
7. Leffler CT, Gozani SN, Cros D. Median neuropathy at the wrist: diagnostic utility of clinical findings and an automated electrodiagnostic device. J Occup. Environ. Med. 2000: 42;398-409
8. Kuo DY, Mallick S, Shen HJ, DeVictoria C, Jones J, Fields AL, Goldberg GL, Runowicz CD, Horwitz SB. Analysis of MDRJ expression in normal and malignant endometrium by reverse transcription-polymerase chain reaction and immunochemistry. Clin. Cancer Res: Dec 1996: 2(12): 1981-92.
9. Kepper D, Konig J, Buchler M. the cananicular multidrug resistance protein, CMRP/MRP2, a novel conjugate export pump expressed in the apical membrane of hepatocytes. Adv. Enzyme Regul :1997: 37:321-33.
10. Kool M, deHaas M Scheffer GL, Scheper RJ, van Eijk, Juijn JA, Baas F, Borst P. Analysis of expression of cMOAT (MRP2), MRP3, MRP4, and MRP5 homologues of the multidrug reststance-associated protein gene (MRP1) in human cancer cell lines. Cancer Res:August 1997: 57(16):3537-47.
11. Horowitz SB, Shen HJ, He L, Dittmar P, Neef R, Chen J, Schubart UK. The microtubule-destabilizing activity of metabalstin(p19) is controlled by phosphorylation. J Bio Vhem: March 1997: 273(35):8129-32.
12. Torres K, Shen HJ, Yang CP, Horowitz SB. Translation of Taxol-induced Raf-1 kinase activation into stathmin and the reorganization of the microtubule cytoskeleton. In preparation, 1999.
13. Thompson CB. Apoptosis in the pathogenesis and treatment of disease. Science:March 1995: 267(5203): 1456-62.
14. Reed JC. Bcl-2 family proteins. Oncogene: December 1998: 17(24):3225-36.
15. Scatena CD, Stewart ZA, Mays D, Tang LJ, Keefer CJ, Leach SD, Pietenpol JA. Mitotic phosphorylation of Bcl-2 during normal cell cycle progression and Taxol-induced growth arrest. J Biol chem.: November 1998:273(46):30777-84.
16. Li F, Ambrosini G, Chu EY, Plescia J, Tognin S, Marchisio PC, Alteri DC. Control of apoptosis and mitotic spindle checkpoint by Survivin. Nature: December 1998: 396(6711): 580-4.

69. Ensign LG, Gehan EA, Kamen DS, Thall PF: An optimal three-stage design for phase II clinical trials. Stat Med 1994;13:1727-36.

# APPENDIX A

**Expected Adverse Events Associated with BMS 247550**

| **Category** | **Adverse Event** |
| --- | --- |
| Allergy / Immunology | Allergic reaction / hypersensitivity (including drug fever) |
| Blood / Bone Marrow | Hemoglobin |
| Blood / Bone Marrow | Leukocytes (total WBC) |
| Blood / Bone Marrow | Neutrophils / Granulocytes (ANC / AGC) |
| Blood / Bone Marrow | Platelets |
| Cardiovascular (Arrhythmia) | Sinus bradycardia |
| Cardiovascular (general) | Edema |
| Cardiovascular (general) | Hypotension |
| Constitutional Symptoms | Fatigue (lethargy, malaise, asthenia) |
| Constitutional Symptoms | Fever (in the sbsence of neutropenia, where neutropenia is defined as AGC < 1.0 x 10^9^/L) |
| Dermatology / Skin | Alopecia |
| Dermatology / Skin | Rash / desquamation |
| Dermatology / Skin | Flushing |
| Dermatology / Skin | Pruritis |
| Dermatology / Skin | Radiation recall reaction 9reaction following chemotherapy in the absence of additional radiation therapy that occurs in a previous radiation port) |
| Gastrointestinal | Anorexia |
| Gastrointestinal | Nausea |
| Gastrointestinal | Vomiting |
| Gastrointestinal | Stomatitis / pharyngitis (oral / pharyngeal mucositis) |
| Gastrointestinal | Constipation |
| Gastrointestinal | Diarrhea patients without a colostomy |
| Gastrointestinal | Diarrhea patients with a colostomy |
| Gastrointestinal | Taste disturbance (dysgeusia) |
| Infection / Febrile Neutropenia | Febrile neutropenia (fever of unknown origin without clinically or microbiologically documented infection (ANC <1.0 x 10^9^/l, fever ≥38.5°C) |
| Infection / Febrile Neutropenia | Infection (documented clinically or microbiologically) with Grade 3 or 4 neutropenia (ANC <1.0 x 10^9^/l) |
| Infection / Febrile Neutropenia | Infection with unknown ANC |
| Infection / Febrile Neutropenia | Infection without neutropenia |
| Musculoskeletal | Muscle weakness 9not due to neuropathy) |

| **Category** | **Adverse Event** |
| --- | --- |
| Neurology | Dizziness/lightheadedness |
| Neurology | Neuropathy – sensory |
| Neurology | Neuropathy-motor |
| Neurology | Insomnia |
| Neurology | Speech impairment (dysphasia or aphasia)Myakgua |
| Neurology | Syncope |
| Pain | Arthralgia (joint pain) |
| Pain | Myalgia (muscle pain) |
| Pain | Neuropathic pain (e.g.: jaw pain, neurologic pain, phantom limb pain, posst-infectious neuralgia, or other painful neuropathies) |
| Pain | Pain-Other (Specify) |
| Pain | Tumor pain (onset or exacerbation of tumor pain due to treatment) |
| Pulmonary | Cough |
| Pulmonary | Dyspnea (shortness of breath) |
| Pulmonary | Hypoxia |
| Pulmonary | Hiccoughs (hiccups, singultus) |
| Pulmonary | Pneumonitis/pulmonary infiltrates |
| Renal/Genitourinary | Urinary retention |

Note: The full list of IMT terms is available on the CTEP home page (<http://ctep.info.nih.gov/CtepInformatics/IMT.htm)>.

**A-2**

# APPENDIX B

**Performance Status Criteria**

| **ECOG Performance Status Scale** | | **Karnofsky Performance Scale** | |
| --- | --- | --- | --- |
| Grade | Descriptions | Percent | Description |
| 0 | Normal activity. Fully active, able to carry on all pre-disease performance without restriction. | 100 | Normal, no complaints, no evidence of disease. |
|  |  | 90 | Able to carry on normal activity; minor signs or symptoms of disease. |
| 1 | Symptoms, but ambulatory. Restricted in physically strenuous activity, but ambulatory and able to carry out work of a light or sedentary nature (e.g., light housework, office work). | 80 | Normal activity with effort; some signs or symptoms of disease. |
|  |  | 70 | Cares for self, unable to carry on normal activity or to do active work. |
| 2 | In bed <50% of the time. Ambulatory and capable of all self-care, but unable to carry out any work activities. Up and about more than 50% of waking hours. | 60 | Requires occasional assistance, but is able to care for most of his/her needs. |
|  |  | 50 | Requires considerable assistance and frequent medical care. |
| 3 | In bed >50% of the time. Capable of only limited self-care, confined to bed or chair more than 50% of waking hours. | 40 | Disabled, requires special care and assistance. |
|  |  | 30 | Severely disabled, hospitalization indicated. Death not imminent. |
| 4 | 100% bedridden. Completely disabled. Cannot carry on any self-care. Totally confined to bed or chair. | 20 | Very sick, hospitalization indicated. Death not imminent. |
|  |  | 10 | Moribund, fatal processes progressing rapidly. |
| 5 | Dead. | 0 | Dead. |

**B-1**

**A**PPENDIX C

Specimen Collection Instructions

**SAMPLING REQUIREMENTS - MOLECULAR STUDIES**

**PRE-THERAPY- (0h cycles 1 and 2)**

1) Peripheral blood mononuclear cells (as cytospins).

Blood is collected into two labeled CPT tubes (Becton Dickinson). Samples are immediately centrifuged at 1000G for 30 minutes and the PBMCs that migrate below the plasma, and above the interface are collected. Alternative methods for harvesting PBMCs are acceptable.

The PBMC pellets are washed twice with 0.5ml 5% FBS in PBS containing 0.02% NaN_3_. Washed PBMCs are gently resuspended in 0.5ml of 5% FBS in PBS, and the cell number determined from ~0.1 – 0.2 ml of this cell suspension (depending on the concentration of cells), using either a haemocytometer, or a coulter counter. Prepare a cell suspension containing approximately 0.5 X 10^6^ cells/ml. This will be used to prepare cytospins.

(A)Label 10 glass slides (Fisherbrand), and pre-wet each slide by centrifugation at maximum speed for 5 minutes with 50μl of 5% FBS/PBS.

(B)Add 100μl of cell suspension to each cytofunnel and centriguge at 1000rpm for 10 minutes.

Carefully remove each slide and allow to air dry. Fix the slides in 100% methanol for 5 minutes at -20°C. Air dry and store at 4°C.

(C)Store fixed slides in a tightly sealed container and ship at ambient temperature. Take precautions to ensure that the slides will not break during shipping. Notify personnel in SB Horwitz laboratory (this should be done as soon as patients are scheduled for drug infusion): Arrange collection of slides.

SHIPPING ADDRESS: Dr. Hayley McDaid

Golding 201

Dept of Molecular Pharmacology

Albert Einstein College of Medicine

1300 Morris Pk Ave

Bronx NY 10461 USA

Tel: 718-430-2192 / 2191

Fax: 718-430-8959

E-Mail: [mcdaid@aecom.yu.edu](mailto:shorwitz@aecom.yu.edu)

Please, e-mail or fax the shipping details, including the tracking number, at the time of shipping.

2) Peripheral blood mononuclear cells (as pellet immersed inRNA Later^TM^)

PBMCs that are left over from (1) should be resuspended in 5X volumes of RNA Later™, (Ambion). Samples should be stored at 4°C overnight, and shipped at ambient temperature. In the event that refrigeration is not possible, samples should be placed on ice for a few hours prior to shipping.

Notify personnel in SB Horwitz laboratory (this should be done as soon as patients are scheduled for drug infusion): Arrange collection of pellets, or same‑day shipment.

Samples should be shipped at ambient temperature.

SHIPPING ADDRESS: Dr. Hayley McDaid

Golding 201

Dept of Molecular Pharmacology

Albert Einstein College of Medicine

1300 Morris Pk Ave

Bronx NY 10461 USA

Tel: 718-430-2192 / 2191

Fax: 718-430-8959

E-Mail: [mcdaid@aecom.yu.edu](mailto:shorwitz@aecom.yu.edu)

Please, e-mail or fax the shipping details, including the tracking number, at the time of shipping.

3) Peripheral Blood Mononuclear Cells (WHOLE BLOOD).

In the event that PBMCs CANNOT be purified from whole blood (i.e 1 and 2 above cannot be completed), collect whole blood into labeled CPT tubes (Becton Dickinson) 2 X 10mL tubes), and SHIP IMMEDIATELY to

SHIPPING ADDRESS: Dr. Hayley McDaid

Golding 201

Dept of Molecular Pharmacology

Albert Einstein College of Medicine

1300 Morris Pk Ave

Bronx NY 10461 USA

Tel: 718-430-2192 / 2191

Fax: 718-430-8959

E-Mail: [mcdaid@aecom.yu.edu](mailto:shorwitz@aecom.yu.edu)

Please, e-mail or fax the shipping details, including the tracking number, at the time of shipping. Complete the details of Blood sampling form (next page) and fax to the laboratory.

Laboratory personnel **MUST** be notified in ADVANCE of the SHIPMENT (24hr before), and the shipment MUST be received within 1-2 hrs, to preserve the integrity of the samples.

DETAILS OF BLOOD SAMPLING ON THE BMS 247550 STUDY

*This form should be used only for samples being shipped to the Horwitz lab for molecular analysis

Patent Name:

Date of Infusion:

Time of Infusion:

Cycle #:

Sampling Time: Baseline Time drawn:

1 hour Time drawn:

24 hours Time drawn

Gauge Needle used:

Vacutainer / Syringe (circle one)

Transport to lab (time deposited to lab):

At room temperature / on wet ice / on dry ice

Peripheral vein / central venous catheter / Picc line

COMMENTS:

4) Tumor biopsies or fine needle aspirates from patients with cutaneous or subcutaneous lesions that area easily accessible for biopsy. No invasive measures will be taken to obtain biopsies. The biopsies will either be a fine needle aspiration or punch biopsy performed in the physician’s office after written informed consent is obtained. No more than three passes with the aspiration or biopsy needle will be done.

- Preparation of slides for Immunohistochemistry (this must be carried out IMMEDIATELY after harvesting, **BEFORE** the sample is immersed in RNA Later™). Prepare 8-10 slides per timepoint.

From biopsy: Imprint the cut tumor surface onto labeled Superfrost plus slides (Fisherbrand): Air dry and fix in 100% methanol for 5 minutes at -20°C. Air dry and store at 4°C.

From fine needle aspirate: Dispense a small aliquot of the aspirate onto Superfrost slides (≈0.5 X 10^5^ cells): Air-dry and fix as for biopsy. Store fixed slides in a tightly sealed container and ship at ambient temperature. Take precautions to ensure that the slides will not break during shipping.

- Tumor biopsy (core biopsy), or fine needle aspirate immersed in RNA Later

Use the residual tumor material from the preparation of slides.

Immerse sample in 5X volumes of RNA Later™, (Ambion). Store at 4°C overnight, and ship at ambient temperature. In the event that refrigeration is not possible, place samples on ice for a few hours prior to shipping.

SHIP SLIDES AND TUMOR MATERIAL AT AMBIENT TEMPERATURE:

SHIPPING ADDRESS: Dr. Hayley McDaid

Golding 201

Dept of Molecular Pharmacology

Albert Einstein College of Medicine

1300 Morris Pk Ave

Bronx NY 10461 USA

Tel: 718-430-2192 / 2191

Fax: 718-430-8959

E-Mail: [mcdaid@aecom.yu.edu](mailto:shorwitz@aecom.yu.edu)

Please, e-mail or fax the shipping details, including the tracking number, at the time of shipping.

5) Buccal Cells (cycle 1 only)

The patient should rinse their mouth twice with water to remove particulate matter. They should then swirl approximately 5mL of fresh water in their mouths for a few minutes. The sample should be decanted into a sterile labeled tube and centrifuged at 2500 rpm for 2 minutes to pellet the cells. The cells should then be resuspended in 5X volumes of RNA Later™, (Ambion). Store at 4°C overnight, and ship at ambient temperature. In the event that refrigeration is not possible, place samples on ice for a few hours prior to shipping.

SHIP SLIDES AND TUMOR MATERIAL AT AMBIENT TEMPERATURE:

SHIPPING ADDRESS: Dr. Hayley McDaid

Golding 201

Dept of Molecular Pharmacology

Albert Einstein College of Medicine

1300 Morris Pk Ave

Bronx NY 10461 USA

Tel: 718-430-2192 / 2191

Fax: 718-430-8959

E-Mail: [mcdaid@aecom.yu.edu](mailto:shorwitz@aecom.yu.edu)

Please, e-mail or fax the shipping details, including the tracking number, at the time of shipping.

**POSTTHERAPY – At 1 hr and 24hr post drug infusion for cycles 1 and 2**

1) Peripheral blood mononuclear cells (as cytospins).

Blood is collected into labeled CPT tubes (Becton Dickinson). Samples are immediately centrifuged at 1000G for 30 minutes and the PBMCs that migrate below the plasma, and above the interface are collected. Alternative methods for harvesting PBMCs are acceptable. The PBMC pellets are washed twice with 0.5ml 5% FBS in PBS containing 0.02% NaN_3_. Washed PBMCs are gently resuspended in 0.5ml of 5% FBS in PBS, and the cell number determined from ~0.1 – 0.2 ml of this cell suspension (depending on the concentration of cells), using either a haemocytometer, or a coulter counter. Prepare a cell suspension containing approximately 0.5 X 10^6^ cells/ml. This will be used to prepare cytospins.

(A)Label 10 glass slides (Fisherbrand), and pre-wet each slide by centrifugation at maximum speed for 5 minutes with 50μl of 5% FBS/PBS.

(B)Add 100μl of cell suspension to each cytofunnel and centriguge at 1000rpm for 10 minutes.

Carefully remove each slide and allow to air dry. Fix the slides in 100% methanol for 5 minutes at -20°C. Air dry and store at 4°C.

(C)Store fixed slides in a tightly sealed container and ship at ambient temperature. Take precautions to ensure that the slides will not break during shipping. Notify personnel in SB Horwitz laboratory (this should be done as soon as patients are scheduled for drug infusion): Arrange collection of slides.

SHIPPING ADDRESS: Dr. Hayley McDaid

Golding 201

Dept of Molecular Pharmacology

Albert Einstein College of Medicine

1300 Morris Pk Ave

Bronx NY 10461 USA

Tel: 718-430-2192 / 2191

Fax: 718-430-8959

E-Mail: [mcdaid@aecom.yu.edu](mailto:shorwitz@aecom.yu.edu)

Please, e-mail or fax the shipping details, including the tracking number, at the time of shipping.

2) Peripheral Blood Mononuclear Cells (WHOLE BLOOD).

In the event that PBMCs CANNOT be purified from whole blood (i.e 1 above cannot be completed), collect whole blood into labeled CPT tubes (Becton Dickinson) 2 X 10mL tubes), and SHIP IMMEDIATELY to

SHIPPING ADDRESS: Dr. Hayley McDaid

Golding 201

Dept of Molecular Pharmacology

Albert Einstein College of Medicine

1300 Morris Pk Ave

Bronx NY 10461 USA

Tel: 718-430-2192 / 2191

Fax: 718-430-8959

E-Mail: [mcdaid@aecom.yu.edu](mailto:shorwitz@aecom.yu.edu)

Please, e-mail or fax the shipping details, including the tracking number, at the time of shipping. Laboratory personel **MUST** be notified in ADVANCE of the SHIPMENT (24hr before), and the shipment MUST be received within 1-2 hrs, to preserve the integrity of the samples.

4) Tumor biopsies or fine needle aspirates from patients with cutaneous or subcutaneous lesions that area easily accessible for biopsy. No invasive measures will be taken to obtain biopsies. The biopsies will either be a fine needle aspiration or punch biopsy performed in the physician’s office after written informed consent is obtained. No more than three passes with the aspiration or biopsy needle will be done.

- Preparation of slides for Immunohistochemistry (this must be carried out IMMEDIATELY after harvesting, **BEFORE** the sample is immersed in RNA Later™). Prepare 8-10 slides per timepoint.

From biopsy: Imprint the cut tumor surface onto labeled Superfrost plus slides (Fisherbrand): Air dry and fix in 100% methanol for 5 minutes at -20°C. Air dry and store at 4°C.

From fine needle aspirate: Dispense a small aliquot of the aspirate onto Superfrost slides (≈0.5 X 10^5^ cells): Air-dry and fix as for biopsy. Store fixed slides in a tightly sealed container and ship at ambient temperature. Take precautions to ensure that the slides will not break during shipping.

- Tumor biopsy (core biopsy), or fine needle aspirate immersed in RNA Later

Use the residual tumor material from the preparation of slides.

Immerse sample in 5X volumes of RNA Later™, (Ambion). Store at 4°C overnight, and ship at ambient temperature. In the event that refrigeration is not possible, place samples on ice for a few hours prior to shipping.

SHIP SLIDES AND TUMOR MATERIAL AT AMBIENT TEMPERATURE:

SHIPPING ADDRESS: Dr. Hayley McDaid

Golding 201

Dept of Molecular Pharmacology

Albert Einstein College of Medicine

1300 Morris Pk Ave

Bronx NY 10461 USA

Tel: 718-430-2192 / 2191

Fax: 718-430-8959

E-Mail: [mcdaid@aecom.yu.edu](mailto:shorwitz@aecom.yu.edu)

Please, e-mail or fax the shipping details, including the tracking number, at the time of shipping.

**Summary of Sampling Parameters for Molecular Analyses:**

|  | **Cycles 1 and 2** | | |  |
| --- | --- | --- | --- | --- |
|  | Pre (0h) | Post (1h) | Post (24h) | Off Study |
| Collection of PBMCs | X | X | X |  |
| Collection of Buccal cells | X |  |  |  |
| Collection of tumor biopsies / fine needle aspirates | X | X | X | X |

**Specimen Collection Forms**

Phase II Scientific Exploration. Study of Epothilone B Analog

(BMS 247550) in Patients with Metastatic Melanoma

Protocol Number:

NCI Number:

IRB Approval (date):

**Tumor Biopsy Specimen Shipment**

Investigator Site:

Sridhir Mani, M.D. Dr Hayley McDaid, PhD

Montefiore Medical Center Albert Einstein College of Medicine

111 East 210^th^ Street 1300 Morris Park Avenue

Bronx, New York 10467 Bronx, New York 10461

Tel: (718) 920-6578 Tel: (718) 430‑8364

Fax: (718) 798‑6251 Fax: (718) 430-8922,

E‑mail: Smani@prodigy.net E‑mail: shorwitz@aecom.vu.edu

| Patient # | Patient  Initials | Date  Biopsy  Collected | Time of  Collection (e.g.  9am=900 hrs:  4pm= 1600 hrs) | Tumor  Type | Cycle | Day | Dose  mg,/kg | Number of  Samples  Collected* |
| --- | --- | --- | --- | --- | --- | --- | --- | --- |
|  |  |  |  |  |  |  |  |  |
|  |  |  |  |  |  |  |  |  |
|  |  |  |  |  |  |  |  |  |
|  |  |  |  |  |  |  |  |  |
|  |  |  |  |  |  |  |  |  |
|  |  |  |  |  |  |  |  |  |

- Must obtain complete pathology report when available.

Site Comments:

Total Samples Shipped:______________________________________________________________________

Date Shipped:______________________________ I Shipped by:__________________________

Federal Express Tracking Number:_____________________________________________________________

PLEASE FAX THIS TO DR. Hayley McDaid AT (7 18) 430‑8959 ON DAY OF SHIPMENT.

Phase II Scientific Exploration. Study of Epothilone B Analog

(BMS 247550) in Patients with Metastatic Melanoma

Protocol Number:

NCI Number:

IRB Approval (date):

Peripheral Blood Mononuclear Cells for Analysis of Tubulin Polymerization (Whole Blood OR slides / pellet of PBMCs)

Investigator Site:

Sridhir Mani, M.D. Dr. Hayley McDaid, PhD

Montefiore Medical Center Albert Einstein College of Medicine

111 East 210^th^ Street 1300 Morris Park Avenue

Bronx, New York 10467 Bronx, New York 10461

Tel: (718) 920-6578 Tel: (718) 430‑8364

Fax: (718) 798‑6251 Fax: (718) 430-8922,

E‑mail: Smani@prodigy.net E‑mail: shorwitz@aecom.vu.edu

| Patient # | Patient  Initials | Date  Biopsy  Collected | Time of  Collection (e.g.  9am=900 hrs:  4pm= 1600 hrs) | Tumor  Type | Cycle | Day | Dose  mg,/kg | Number of  Samples  Collected* |
| --- | --- | --- | --- | --- | --- | --- | --- | --- |
|  |  |  |  |  |  |  |  |  |
|  |  |  |  |  |  |  |  |  |
|  |  |  |  |  |  |  |  |  |
|  |  |  |  |  |  |  |  |  |
|  |  |  |  |  |  |  |  |  |
|  |  |  |  |  |  |  |  |  |

- Must obtain complete pathology report when available.

Site Comments:

Total Samples Shipped:______________________________________________________________________

Date Shipped:______________________________ I Shipped by:__________________________

Federal Express Tracking Number:_____________________________________________________________

PLEASE FAX THIS TO DR. Hayley McDaid AT (7 18) 430‑8959 ON DAY OF SHIPMENT.

**APPENDIX D**

**ELIGIBILITY CRITERIA**

| Confirmed Stage IV Melanoma |  |
| --- | --- |
| Measurable Disease |  |
| Scans within 4 weeks |  |
| ECOG Performance Status 0-2 |  |
| ANC > 1.5 x 10^9^/l |  |
| Platelets > 100 x 10^9^/l |  |
| Total Bilirubin-normal |  |
| AST/ALT < 2.5 x ULN |  |
| Creatinine < 1.5 x ULN |  |
| No Brain Mets or Stable Brain Mets for at least 6 weeks after whole brain radiation |  |
| Not pregnant |  |
| No peripheral neuropathy |  |
| No uncontrolled medical illnesses |  |
| Signed informed consent |  |

**APPENDIX E**

**SAMPLE DATA COLLECTION FORM**

NYU 00-57 Guidelines for Data Collection

Patient Registration:

After patient has been screened for eligibility and signed an informed consent, Patient Registration and Eligibility Checklist forms will be sent via fax or email to the Coordinating Center. The Coordinating Center will return the Patient Registration form with the assigned patient id number.

A copy of the informed consent together with copies of source documents confirming eligibility will be sent via fax or mail as soon as possible after the patient is on-study.

Data Collection:

An EXCEL spreadsheet template will be sent electronically to each site. The information collected on the spreadsheet is entered into the NCI CDUS web-based application and submitted to the NCI quarterly on the following schedule:

Submission Deadline Period Covered

30 April 01 Jan - 31 Mar

31 July 01 Mar - 30 Jun

31 October 01 Jul - 30 Sep

31 January 01 Oct - 31 Dec

Updated spreadsheets will be sent to the Coordinating Center on a regular basis and at least 2 weeks before each NCI submission deadline.

In addition to the spreadsheet, copies of source documents will be sent via fax or mail to the Coordinating Center when a patient goes off-study or more frequently for responders.

# ON-STUDY REGISTRATION

| NYU 00-57 A Phase II Study of Epothilone B Analog BMS 247550 in Stage IV Malignant Melanoma | | | |
| --- | --- | --- | --- |
| Date Submitted:  Mo / Dy / Yr ___/ ___/ ___ | NCI Protocol #:  **4470** | Patient Initials: | |
| SEX:  [ ] M [ ] F | Date of Birth:  Day/ Mon / Yr  ___/ ____/ ___ | Age (yrs) | Ethnicity: |
| Body Weight (Kg) | | Height (cm) | |
| Method of Payment: | | Postal Code: | |
| Histology:  **Melanoma** | | Performance Status: | |
| Date of Diagnosis:  Mo / Dy / Yr: _____/ _____/ _____ | | Date On-Study:  Mo / Dy / Yr: _____/ _____/ _____ | |
| Prior Therapy  Chemo [ ] Y [ ] N Hormone [ ] Y [ ] N Immuno [ ] Y [ ] N  Radiation [ ] Y [ ] N Surgery [ ] Y [ ] N | | | |

Patient ID Number: _________________ (assigned at Coordinating Center)

Completed By:

Name: ____________________ Signature: ___________________ Date: ___/ ___/ ___
